# Supplementary material for: Synthesis and Characterization of a Two-Station Two-Gate Calix[6]arene-Based [2]Catenane
Source: Molecules. 2025 Feb 6;30(3):732. doi: 10.3390/molecules30030732 (PMC11820616; doi:10.3390/molecules30030732)
Supplement: Supplementary file 1 [file molecules-30-00732-s001.zip › molecules-3377818-supplementary.pdf]

Supporting Material

Synthesis and Characterization of a Two-station Two-gate Calix[6]arene-based [2]Catenane

Margherita Bazzoni,[1] Francesco Rispoli,[1] Sara Venturelli,[1] Gianpiero Cera,[1] and Andrea Secchi[1,*]

[1] Dipartimento di Scienze Chimiche, della Vita e della Sostenibilità Ambientale, Università di Parma, Parco Area delle Scienze 17/A, I-43124 Parma, Italy. E-mail: andrea.secchi@unipr.it

* Correspondence: andrea.secchi@unipr.it; Tel.: +39-0521 905 409.

Table of contents

Characterization of compound **3**	S2
Characterization of compound **7**	S3-4
Characterization of compound **8**	S5
Characterization of semi-axle **11**	S6-7
Characterization of axle **10**	S8-9
Characterization of rotaxanes **12**(*azo-up*) and **12**(*azo-down*)	S10-S18
Characterization of oriented catenane **C3**(*azo-down)*	S19-S22

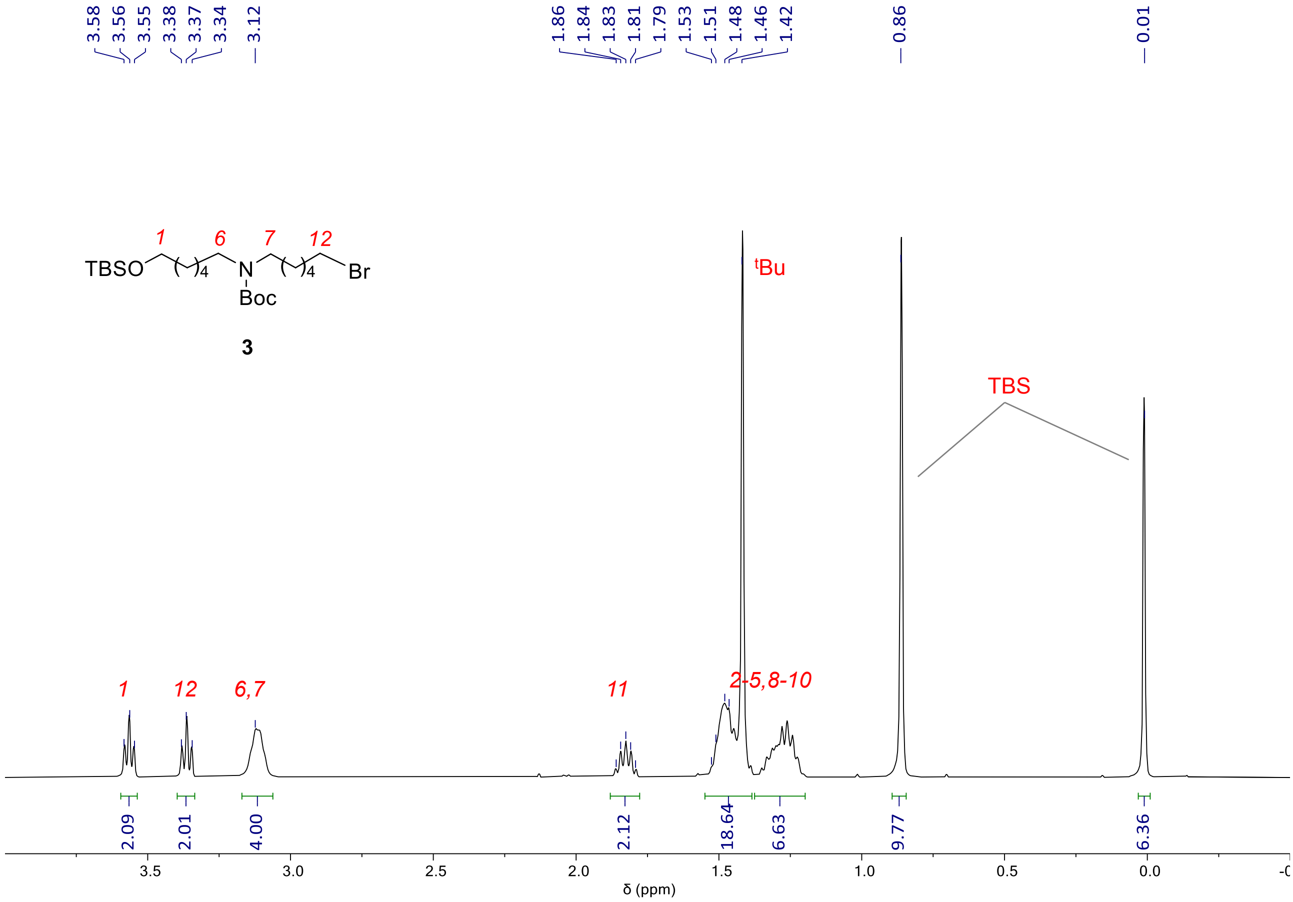

Figure S1. ^{1}H NMR spectrum (300 MHz, $CDCl_3$, 298 K) of compound **3**.

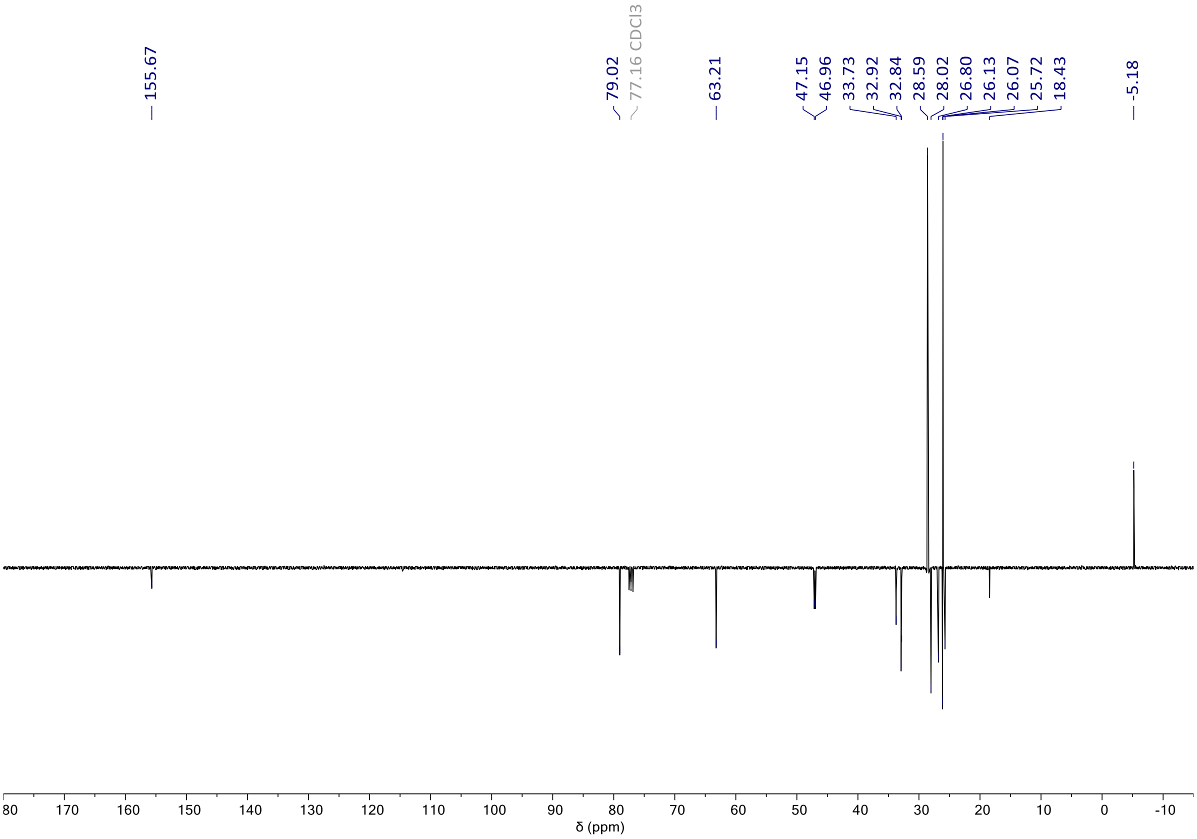

Figure S2. ^{13}C-APT NMR spectrum (100 MHz, $CDCl_3$, 298 K) of compound **3**.

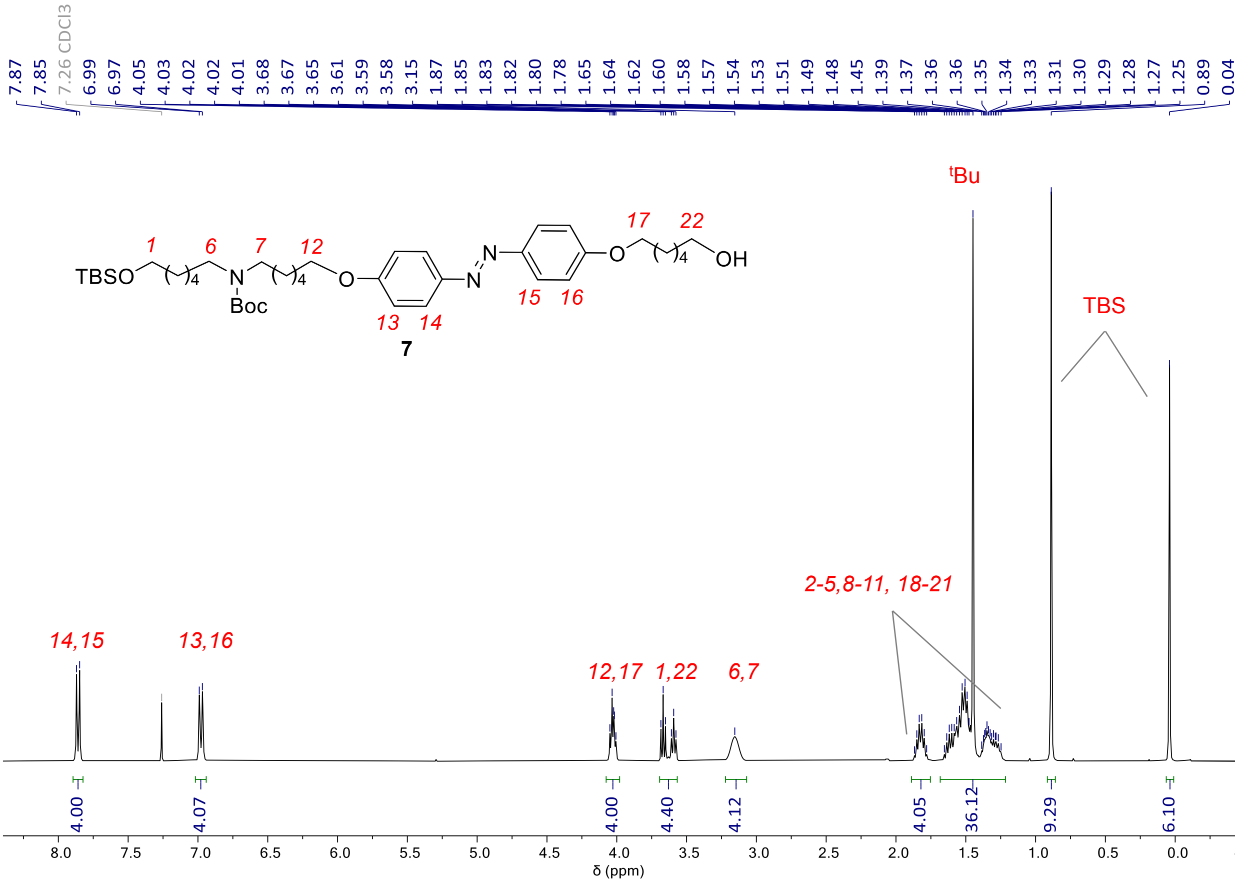

Figure S3. ^1^H NMR spectrum (300 MHz, $CDCl_3$, 298 K) of compound **7**.

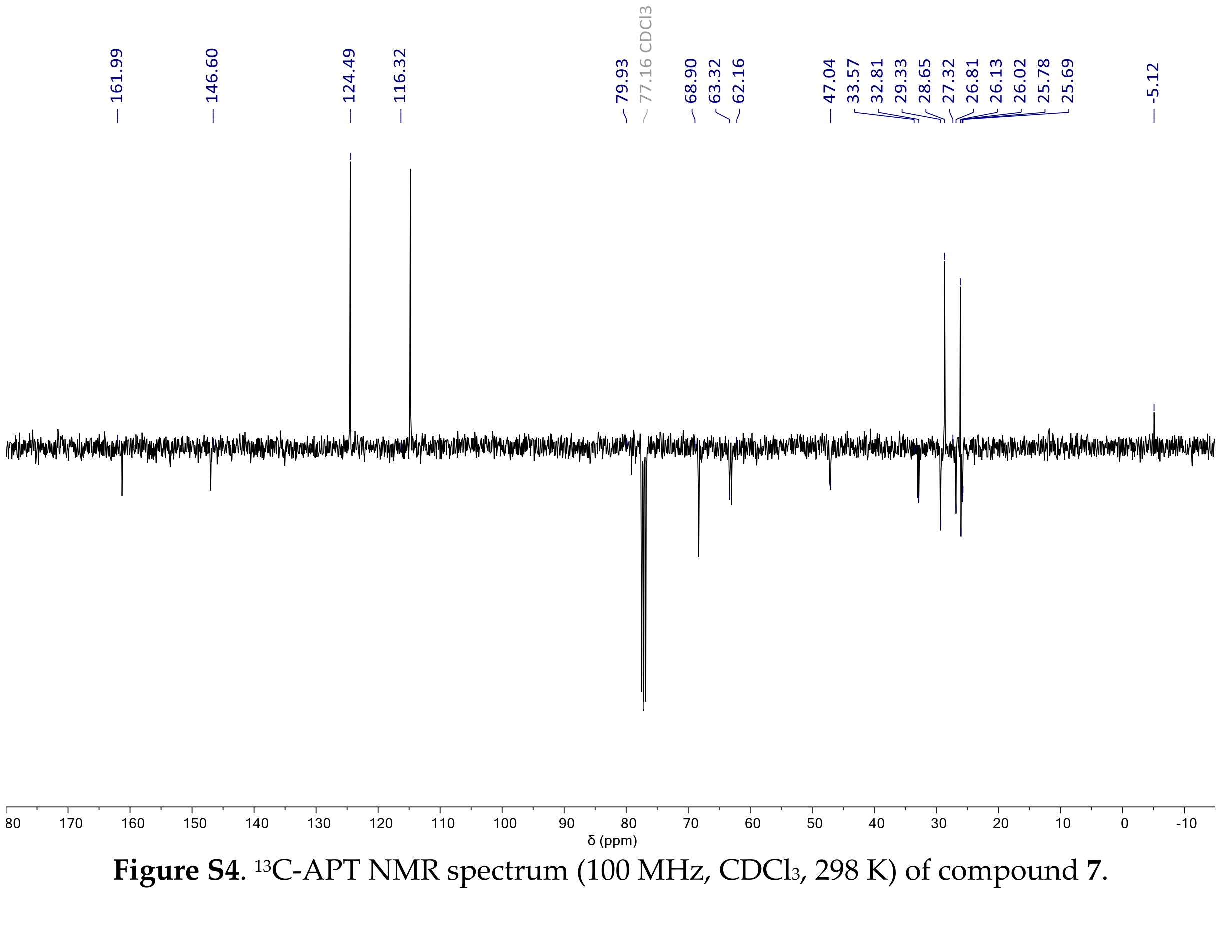

Figure S4. ^13^C-APT NMR spectrum (100 MHz, $CDCl_3$, 298 K) of compound **7**.

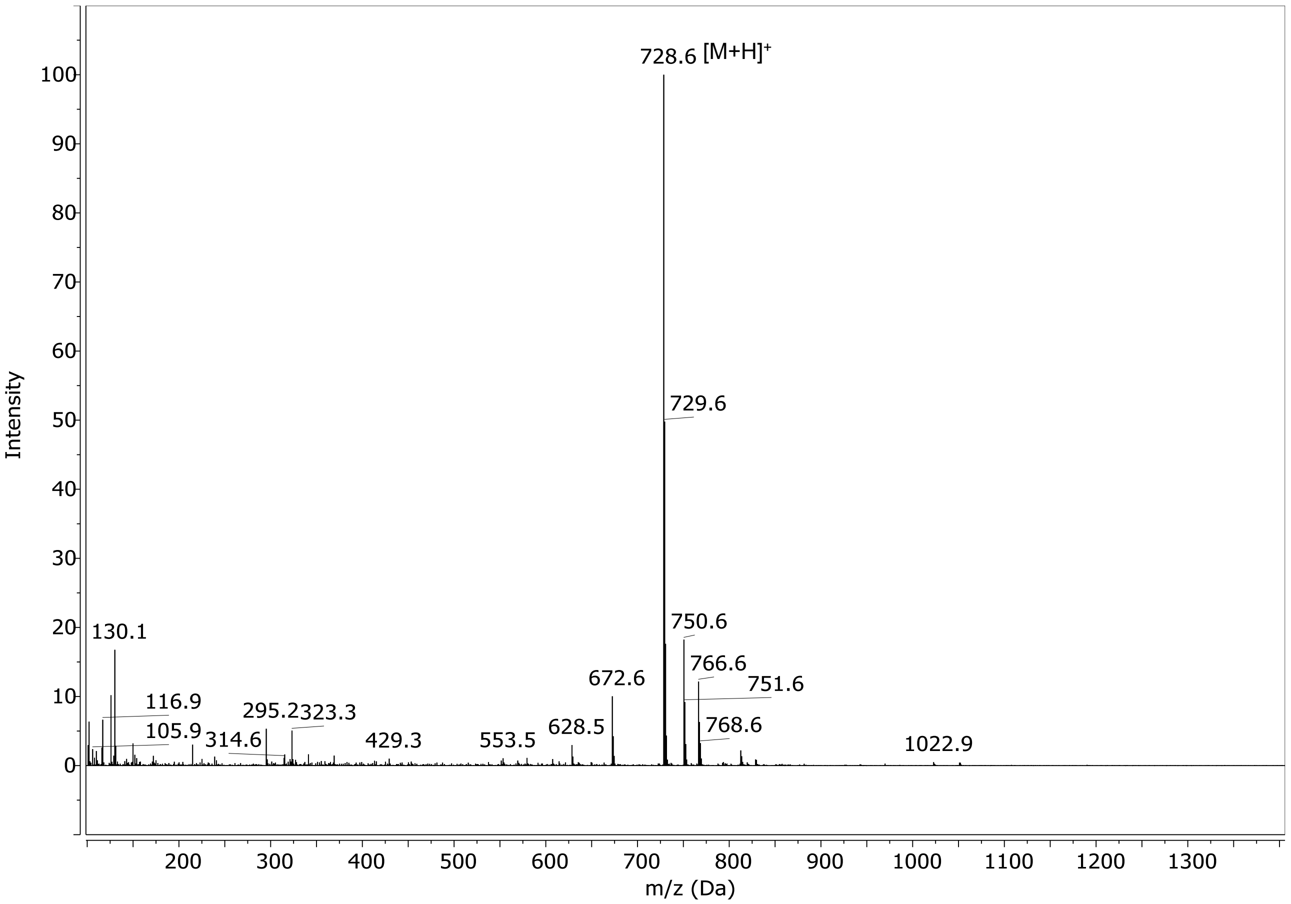

Figure S5. ESI-MS(+) spectrum of compound **7**.

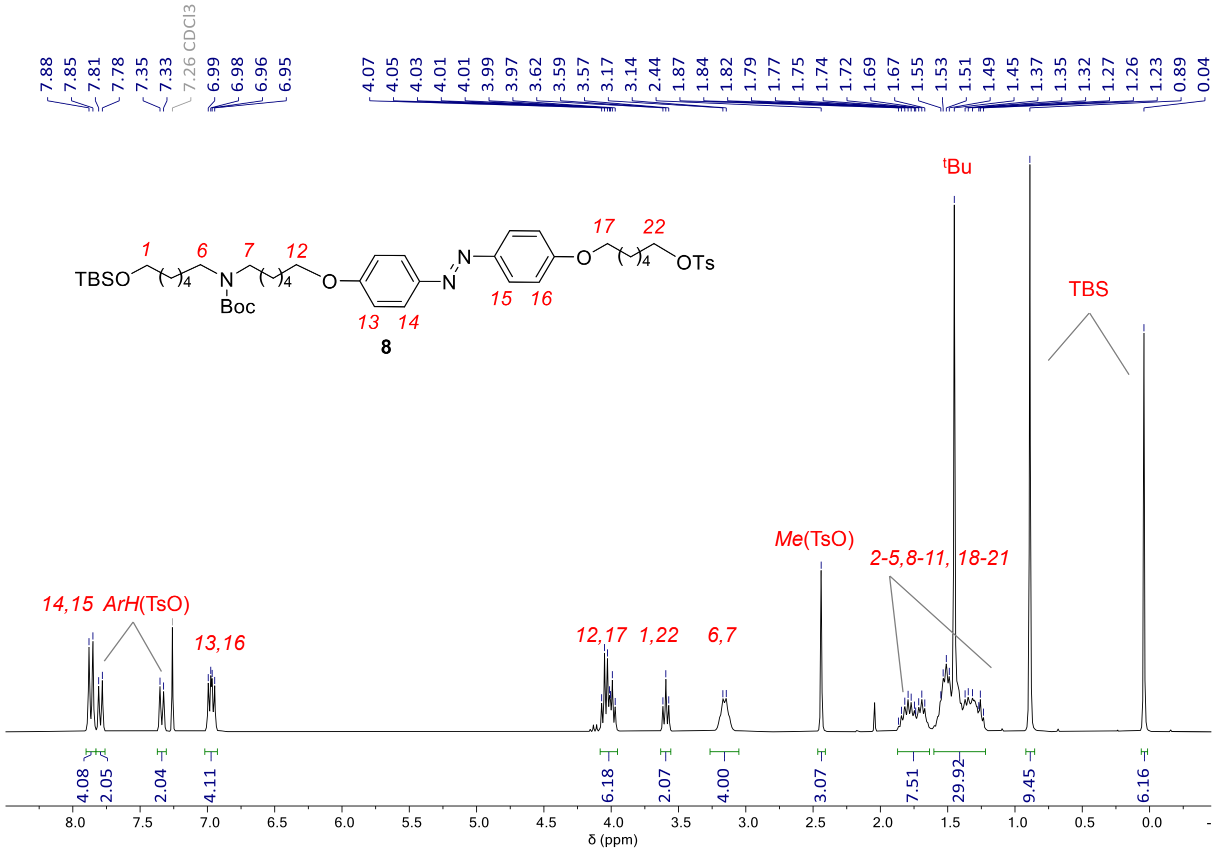

Figure S6. ^{1}H NMR spectrum (300 MHz, $CDCl_3$, 298 K) of compound **8**.

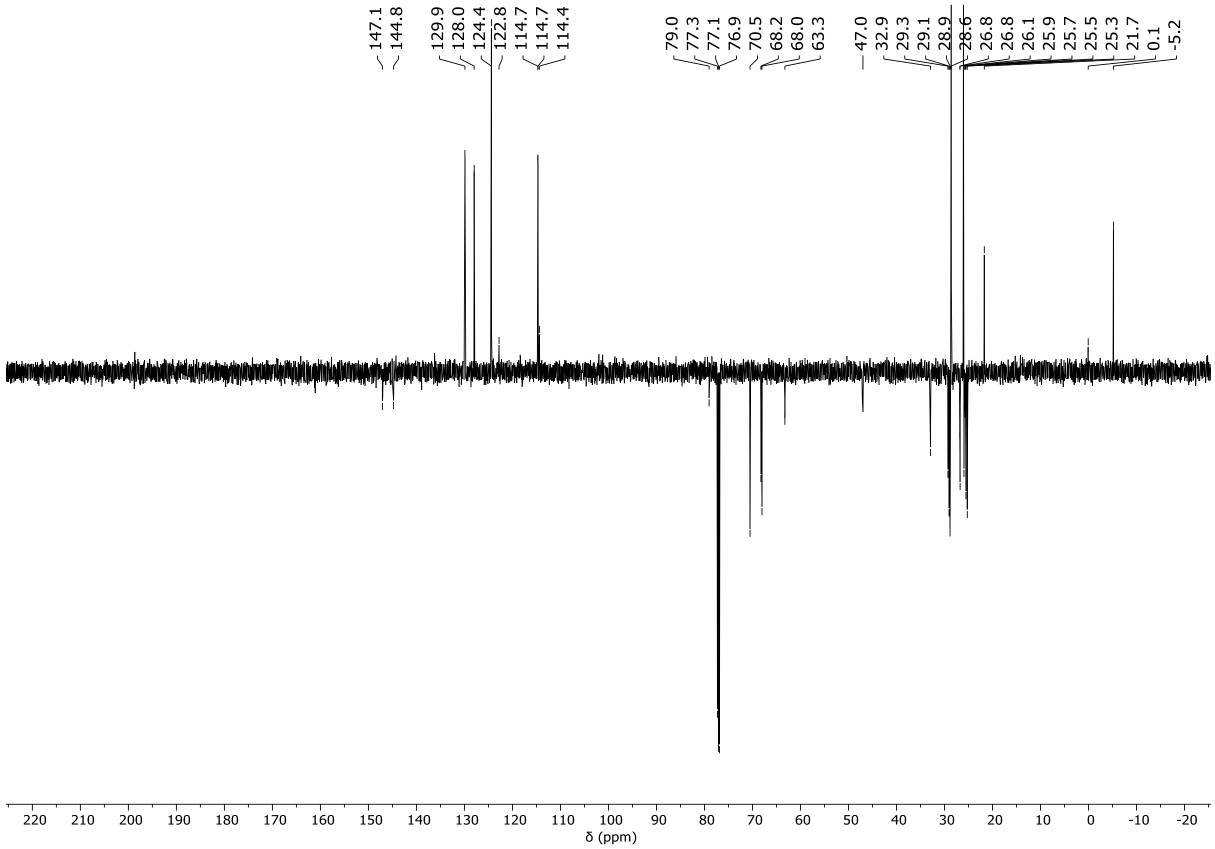

Figure S7. ^{13}C-DEPTQ NMR spectrum (150 MHz, $CDCl_3$, 298 K) of compound **8**.

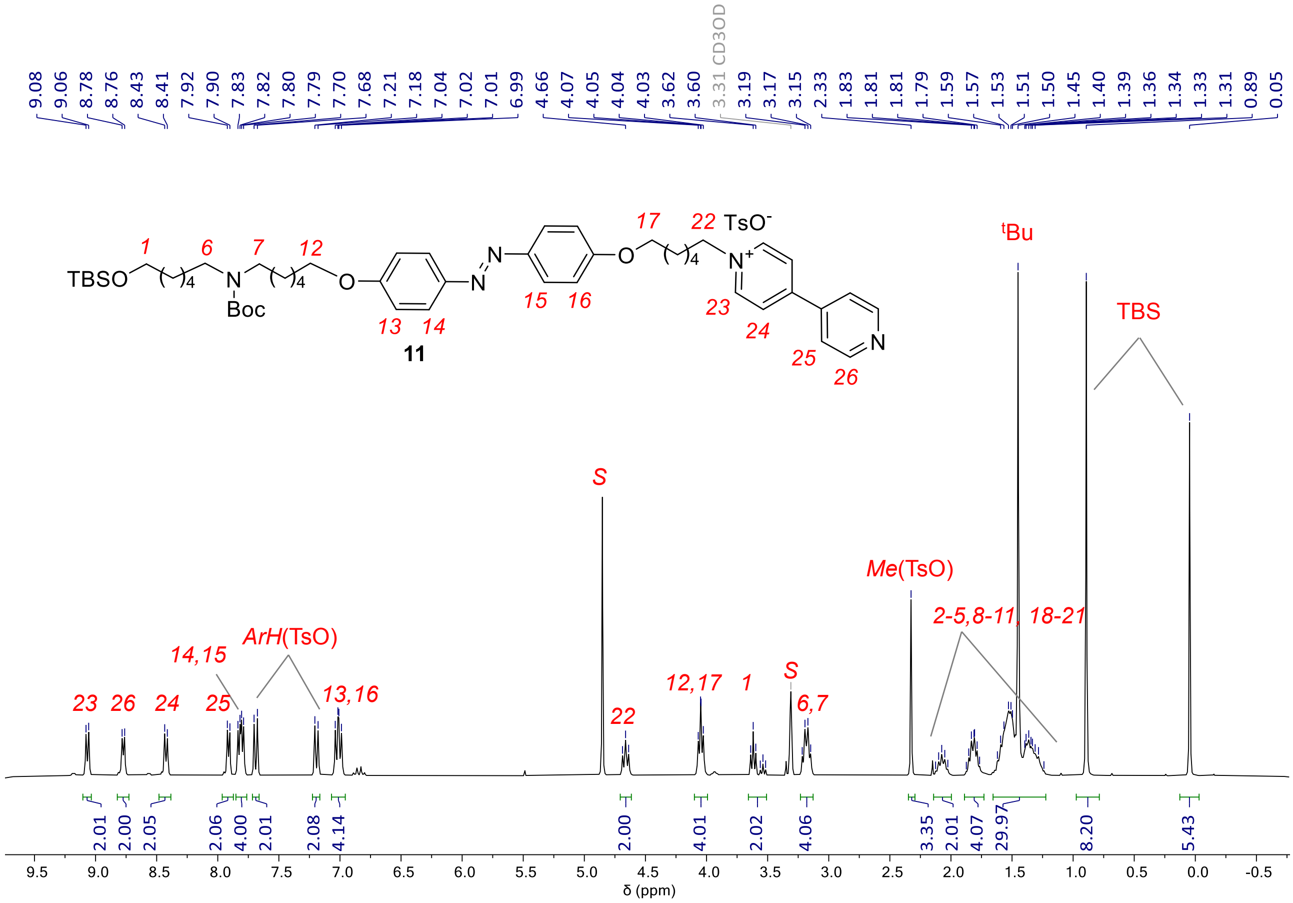

Figure S8. ^{1}H NMR spectrum (300 MHz, CD_3OD, 298 K) of semi-axle **11**.

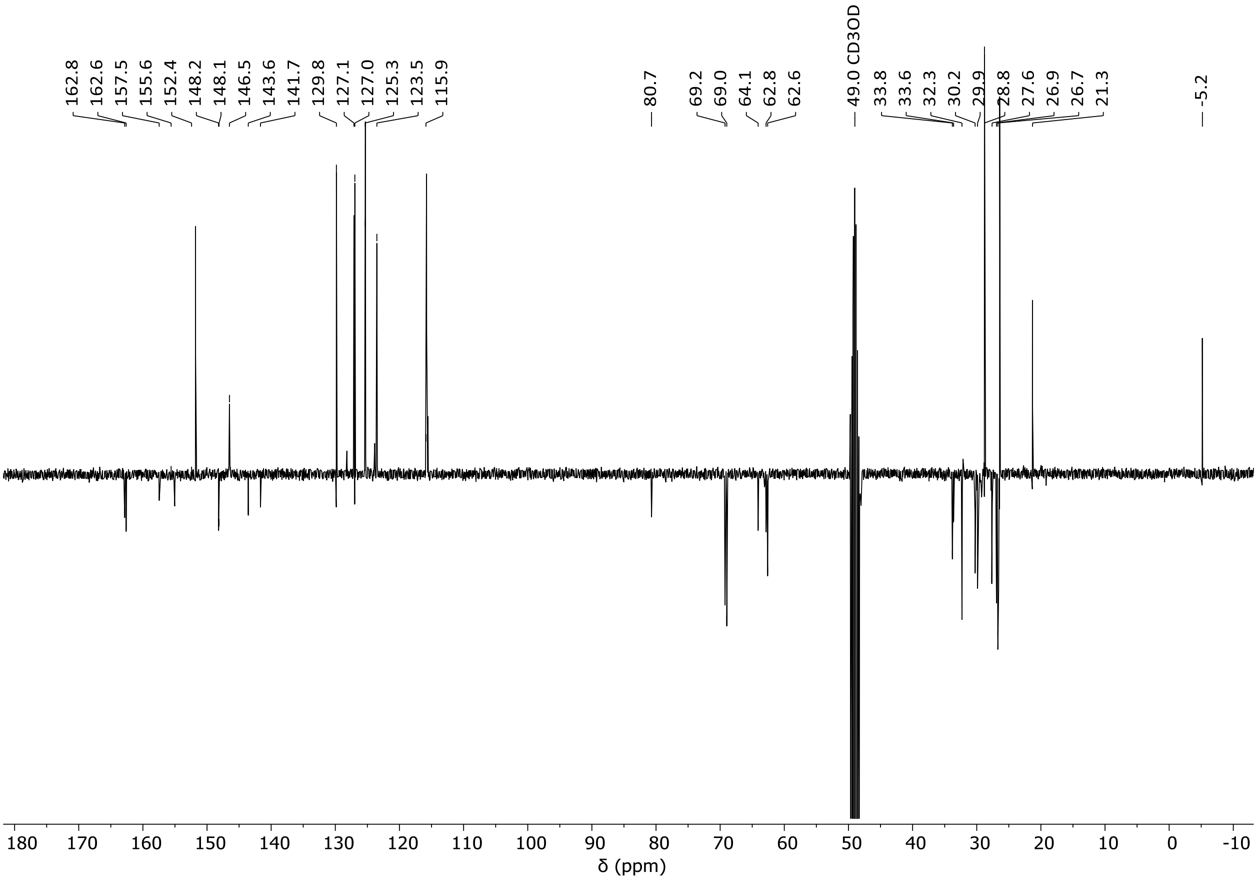

Figure S9. ^{13}C-APT NMR spectrum (100 MHz, CD_3OD, 298 K) of semi-axle **11**.

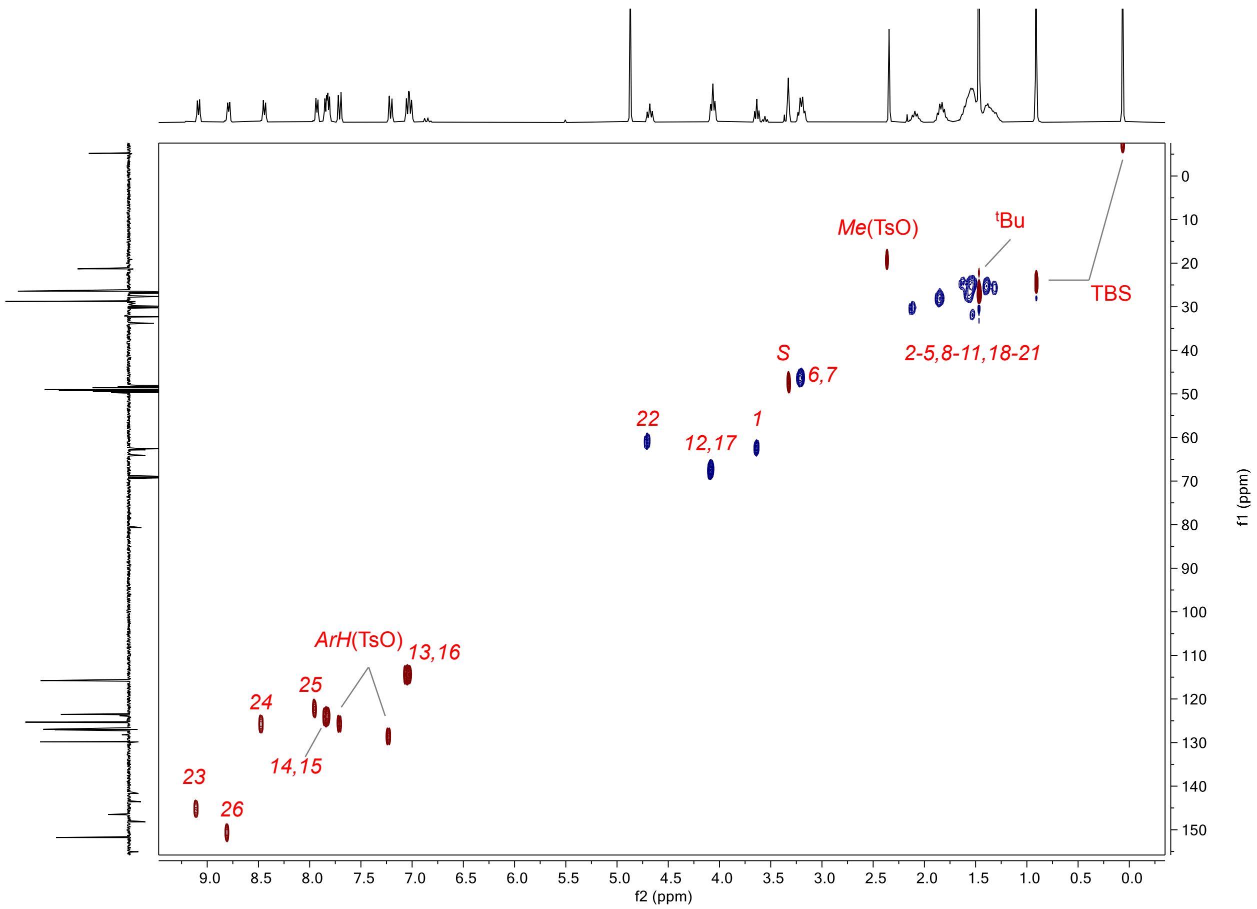

Figure S10. 2D Edited HSQC NMR spectrum (400 MHz, CD_3OD, 298 K) of semi-axle **11** Positive peaks (CH_3 and CH) are shown in red, while negative ones (CH_2) are in blue.

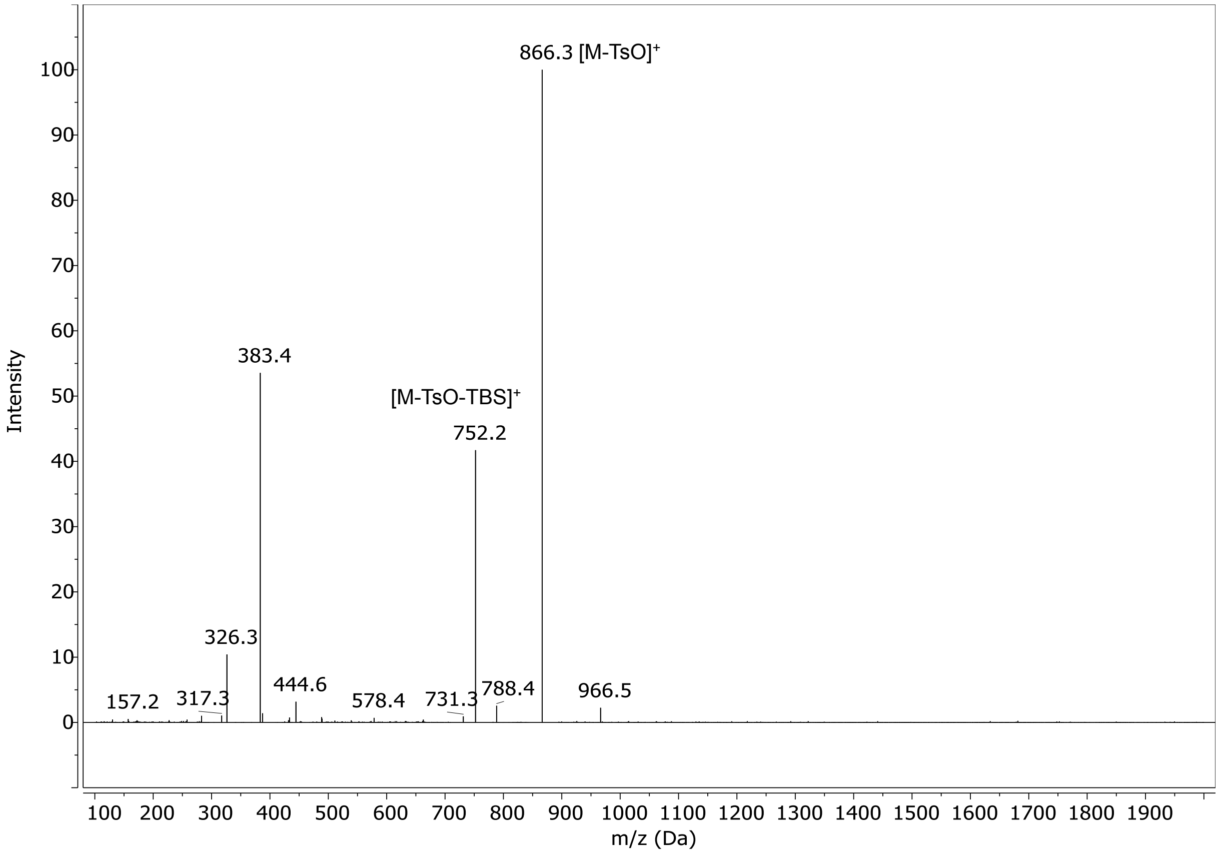

Figure S11. ESI-MS(+) spectrum of compound **11**.

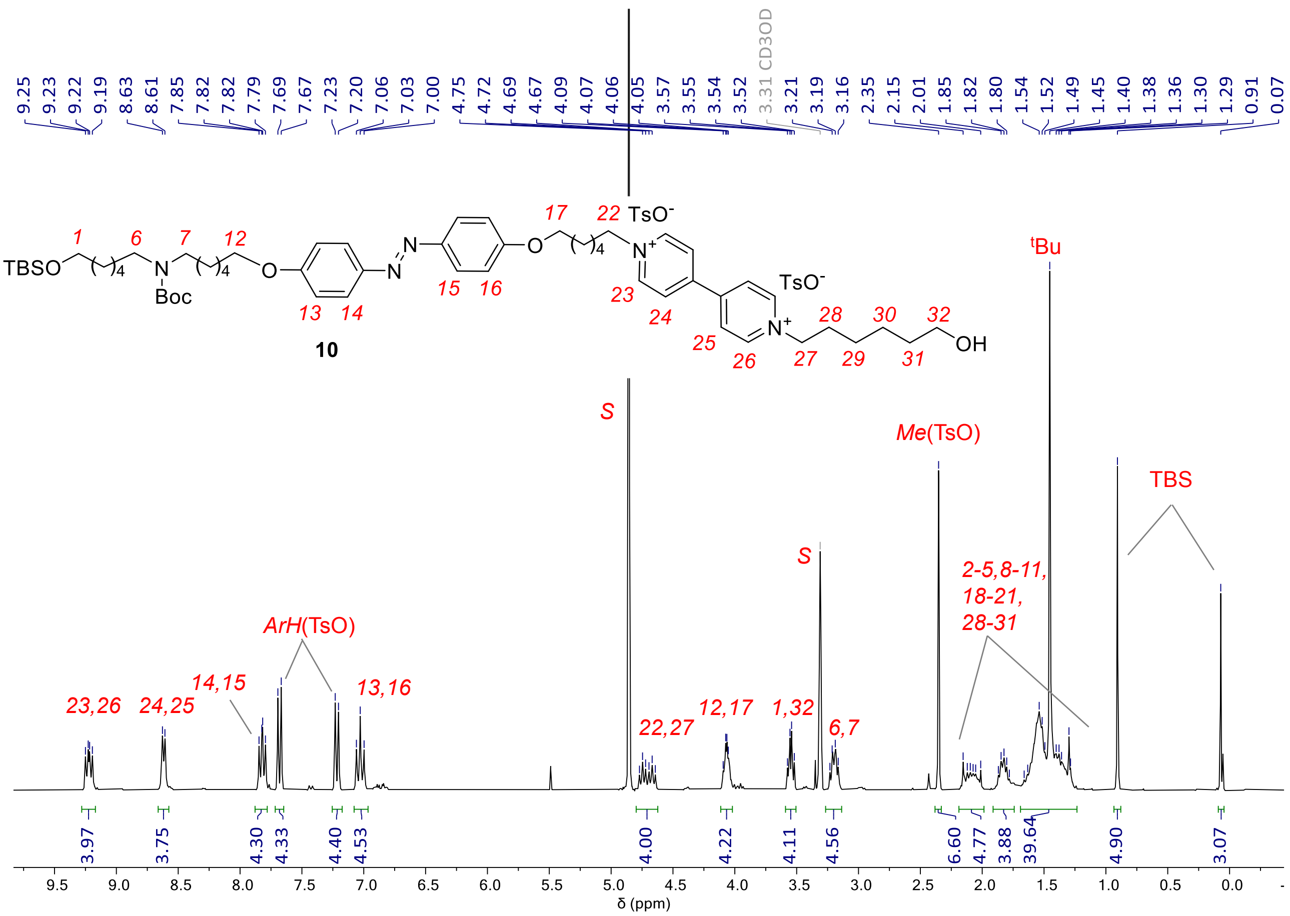

Figure S12. ^{1}H NMR spectrum (300 MHz, CD_3OD, 298 K) of axle **10**.

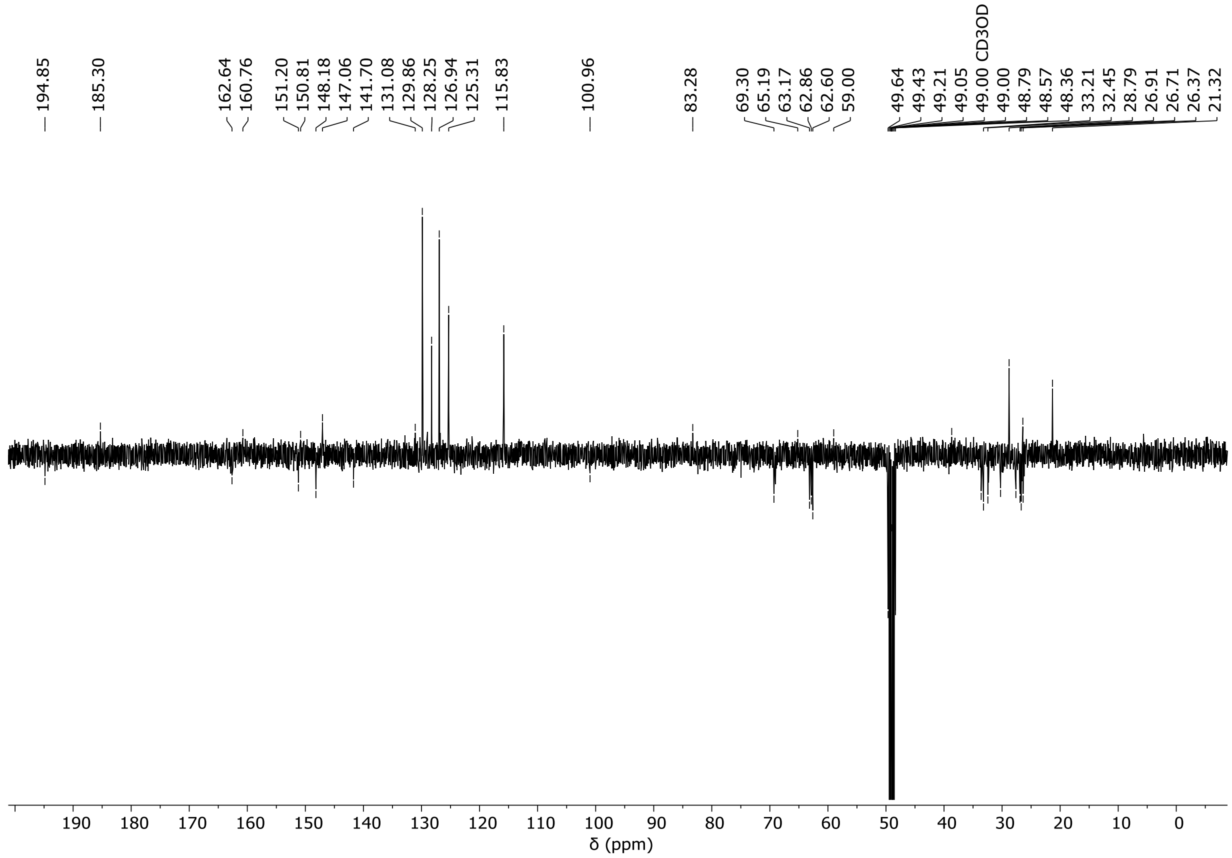

Figure S13. ^{13}C-DEPTQ NMR spectrum (100 MHz, CD_3OD, 298 K) of axle **10**.

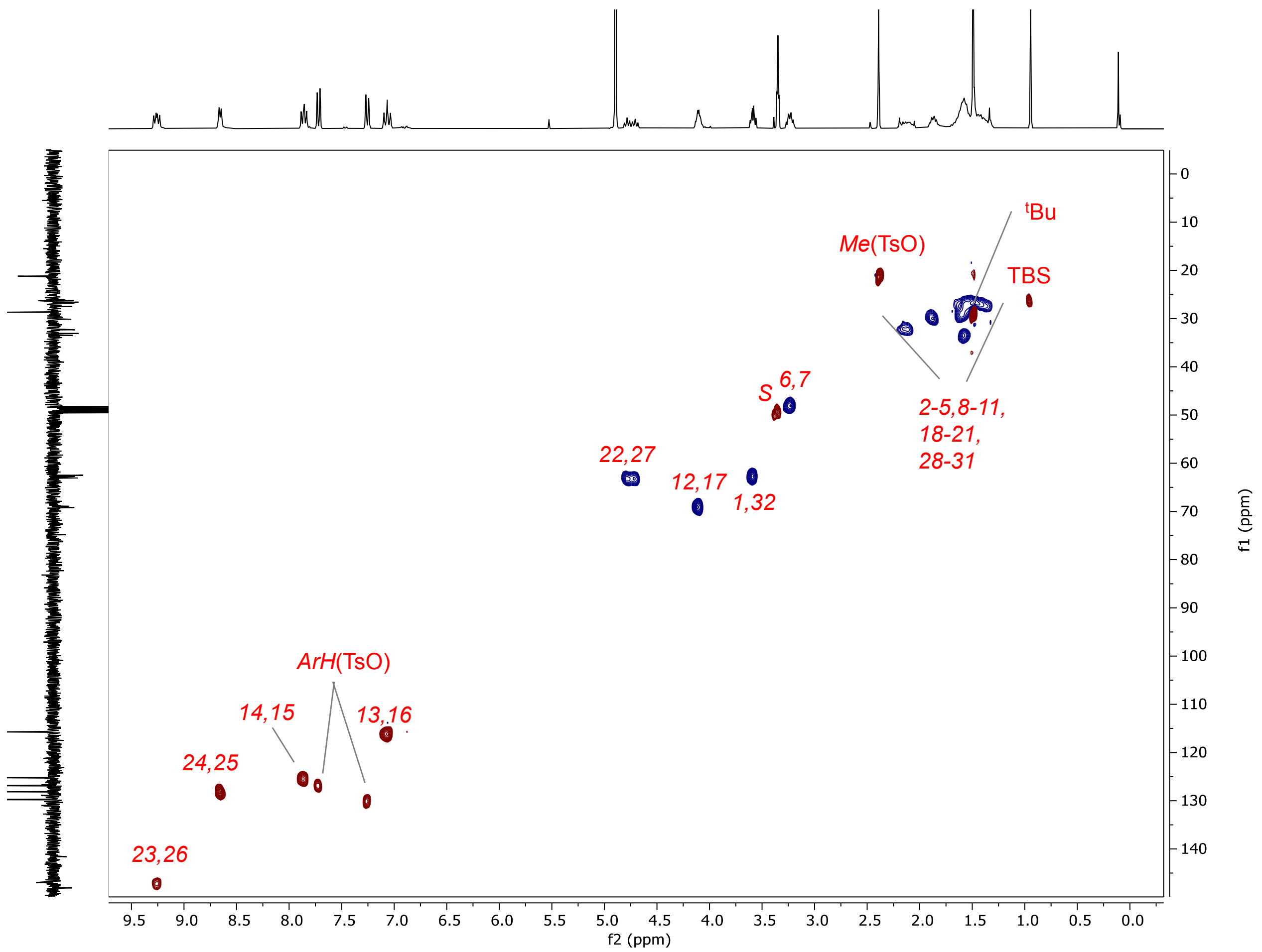

Figure S14. 2D Edited HSQC NMR spectrum (400 MHz, CD_3OD, 298 K) of axle **10** Positive peaks (CH_3 and CH) are shown in red, while negative ones (CH_2) are in blue.

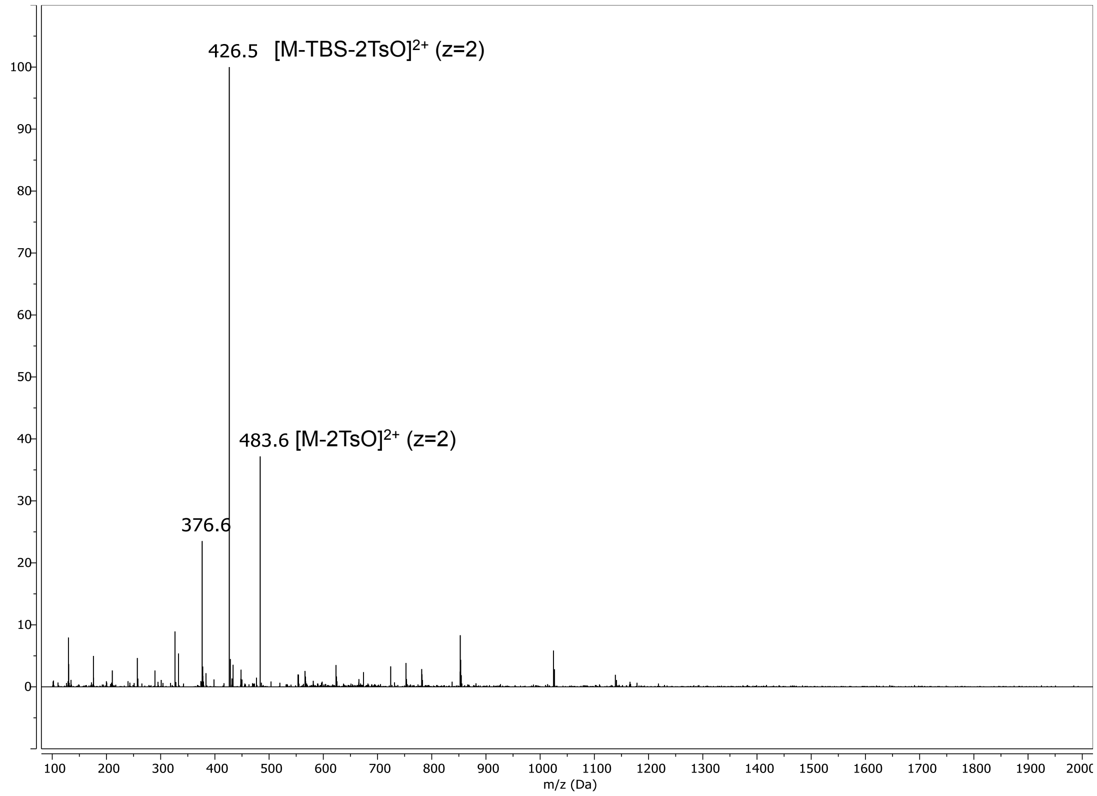

Figure S15. ESI-MS(+) spectrum of axle **10**.

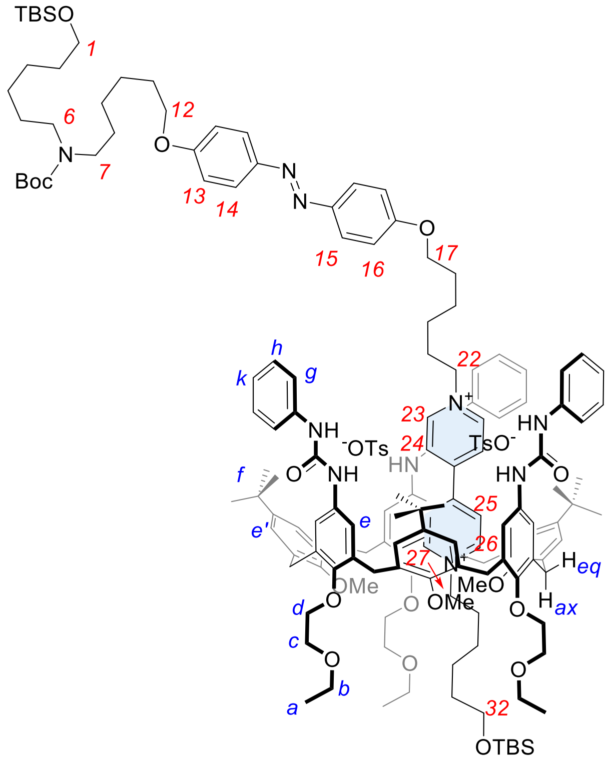

12(*azo-up*)

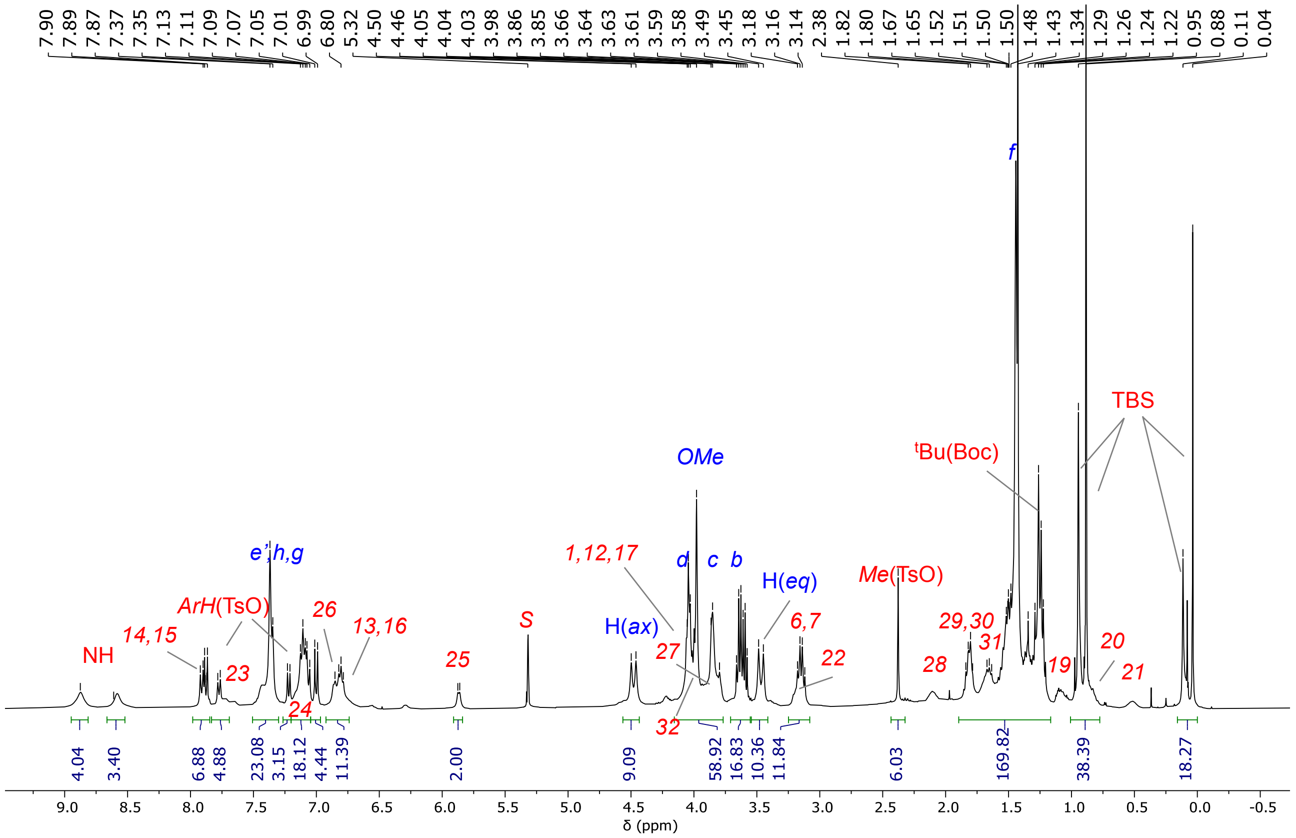

Figure S16. ^{1}H NMR spectrum (400 MHz, CD_2Cl_2, 298 K) of oriented rotaxane **12**(*azo-up*).

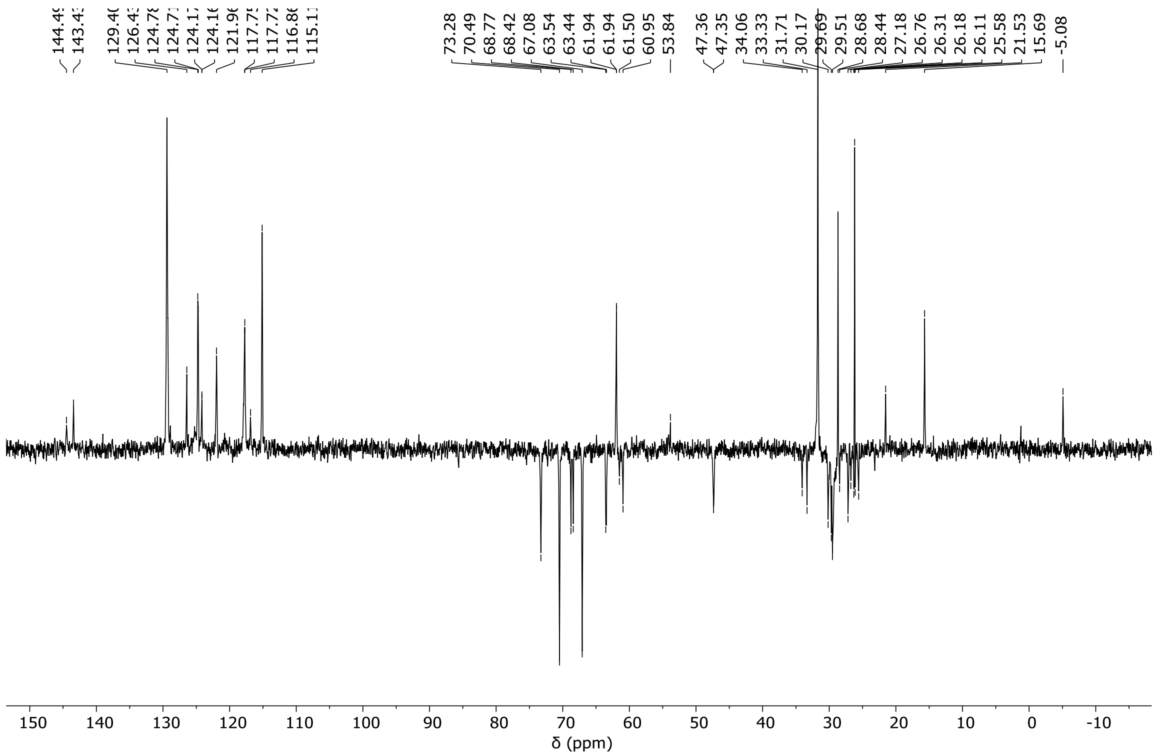

Figure S17. ^{13}C-APT NMR spectrum (100 MHz, CD_2Cl_2, 298 K) of oriented rotaxane **12**(*azo-up*).

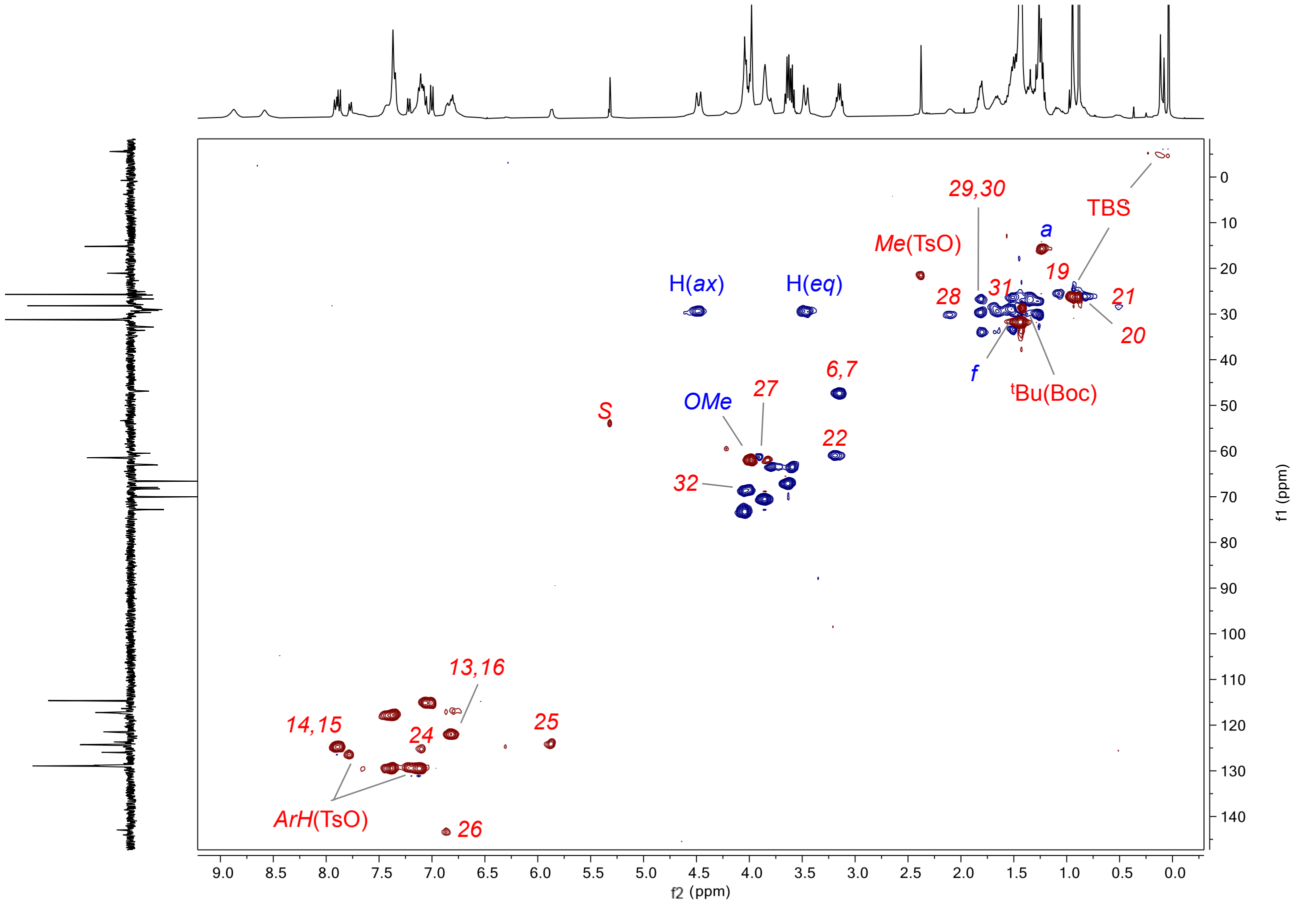

Figure S18. 2D Edited HSQC NMR spectrum (400 MHz, CD_2Cl_2, 298 K) of oriented rotaxane **12**(*azo-up*). Positive peaks (CH_3 and CH) are shown in red, while negative ones (CH_2) are in blue.

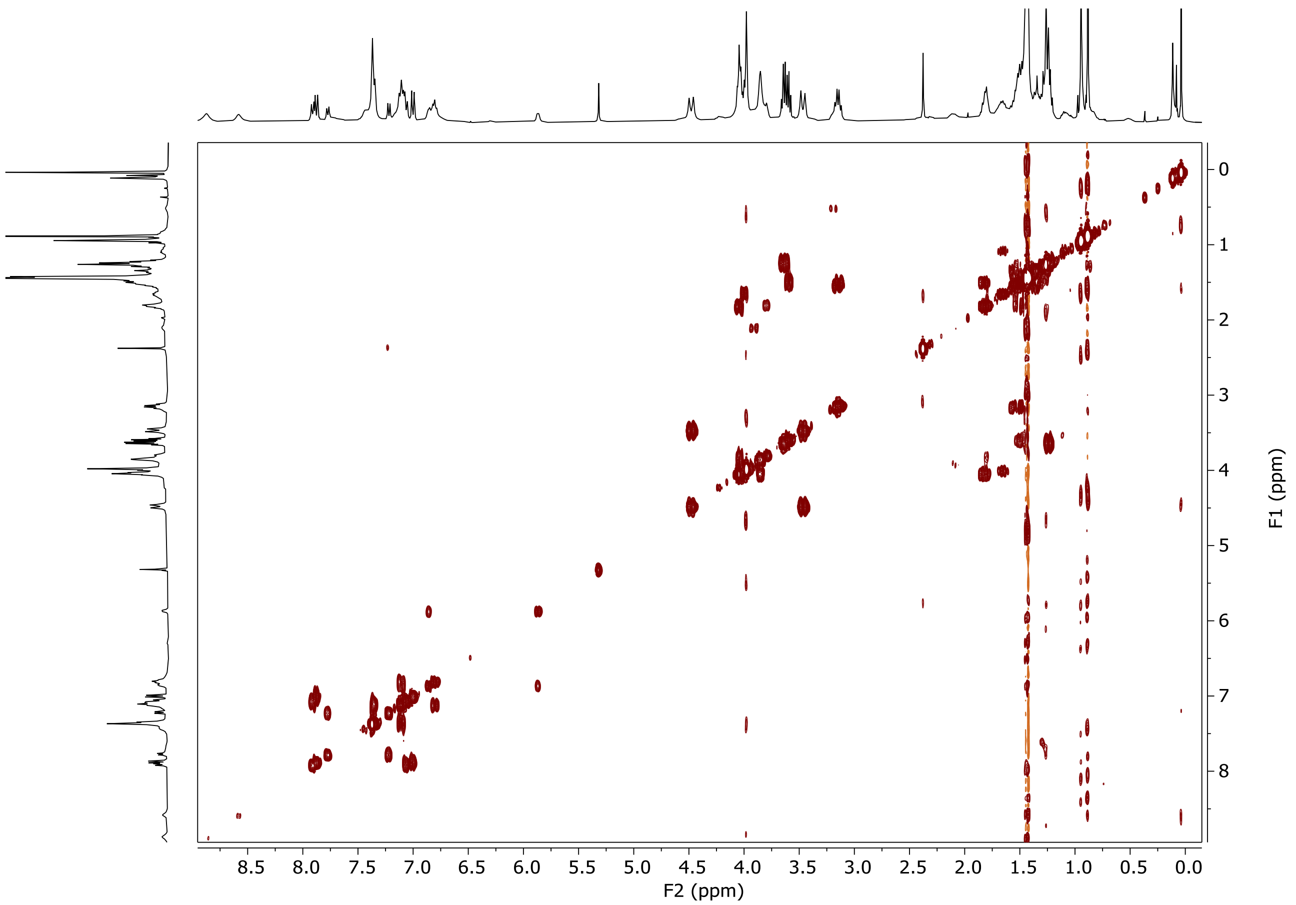

Figure S19. 2D COSY NMR spectrum (400 MHz, CD_2Cl_2, 298 K) of oriented rotaxane **12**(*azo-up*).

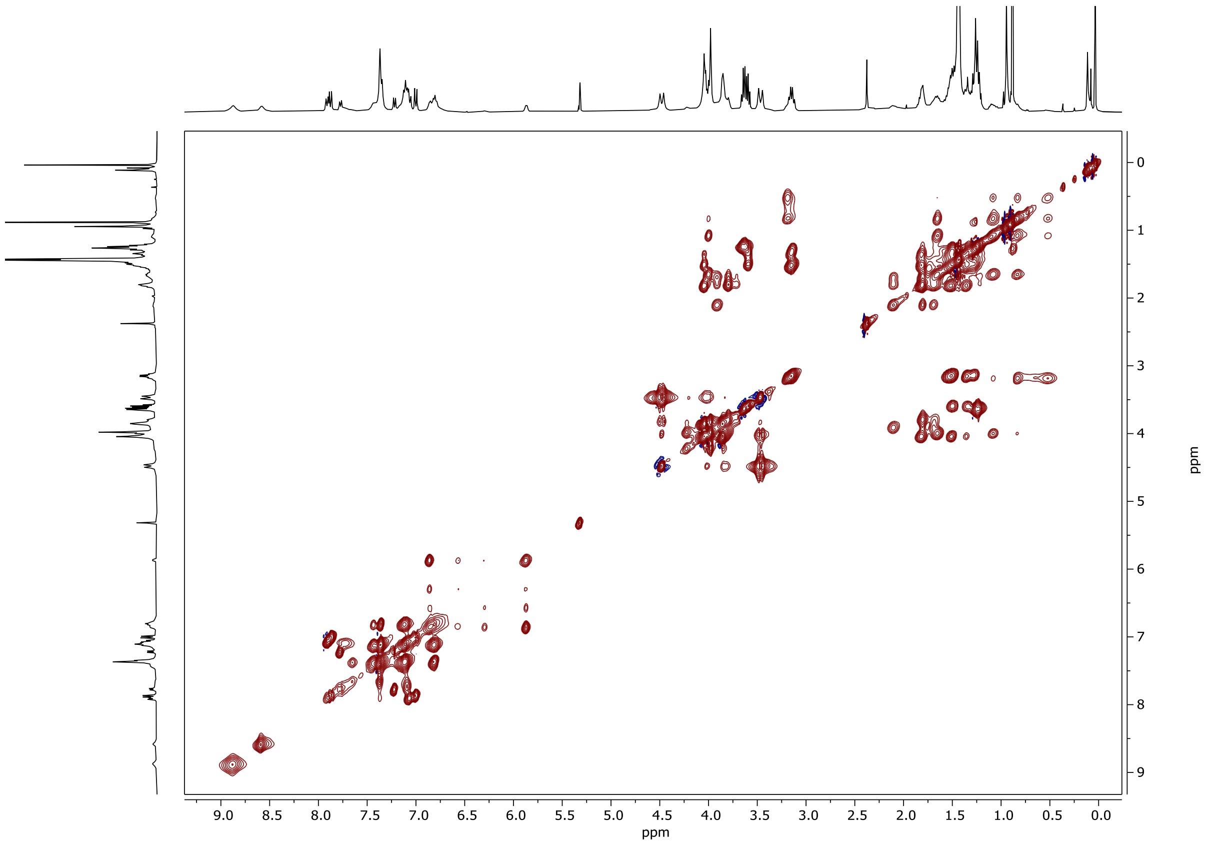

Figure S20. 2D TOCSY NMR spectrum (400 MHz, CD_2Cl_2, 298 K, Mixing time = 0.04 s) of oriented rotaxane **12**(*azo-up*).

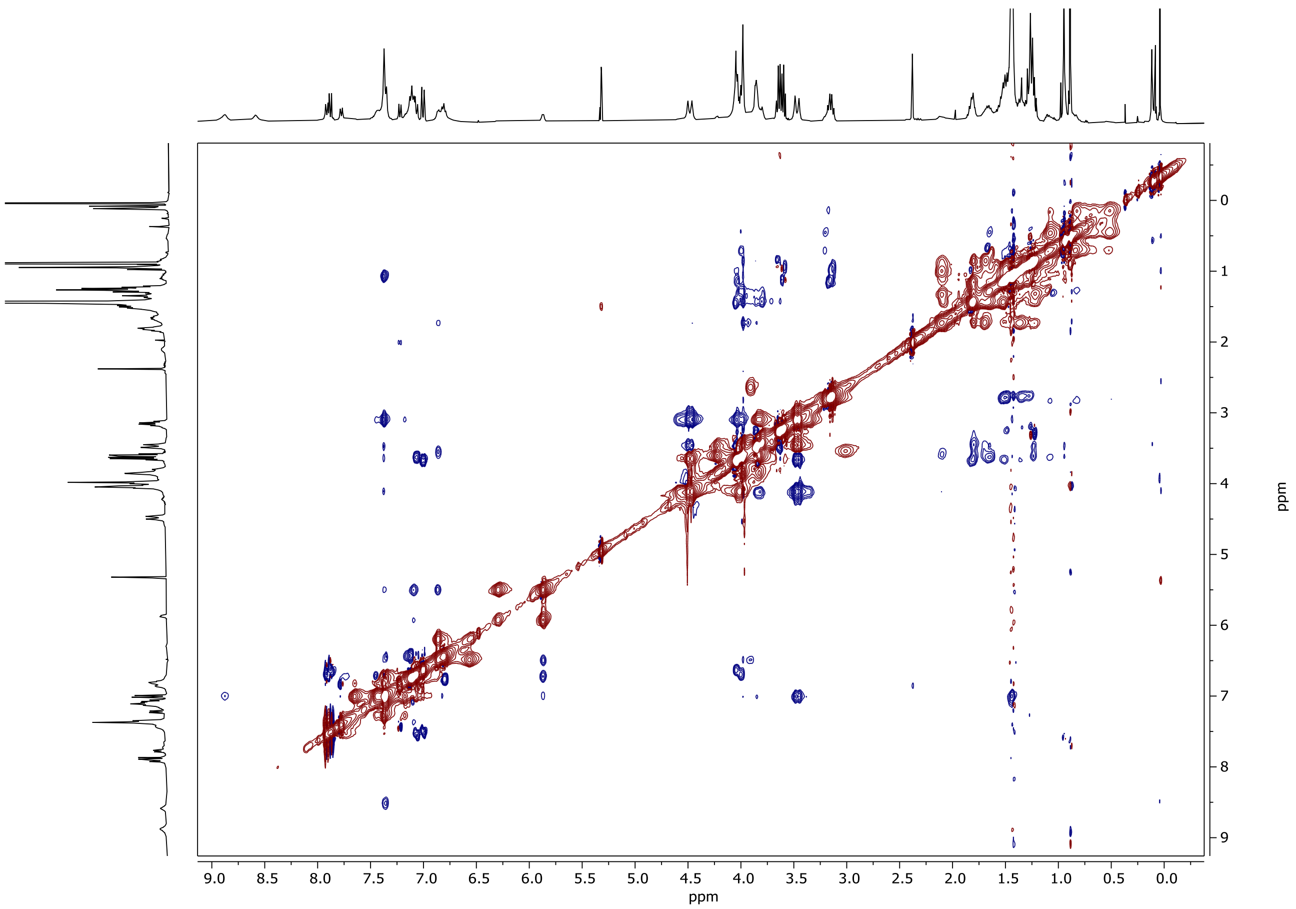

Figure S21. 2D ROESY NMR spectrum (400 MHz, CD_2Cl_2, 298 K, Spin locking = 200 ms) of oriented rotaxane **12**(*azo-up*).

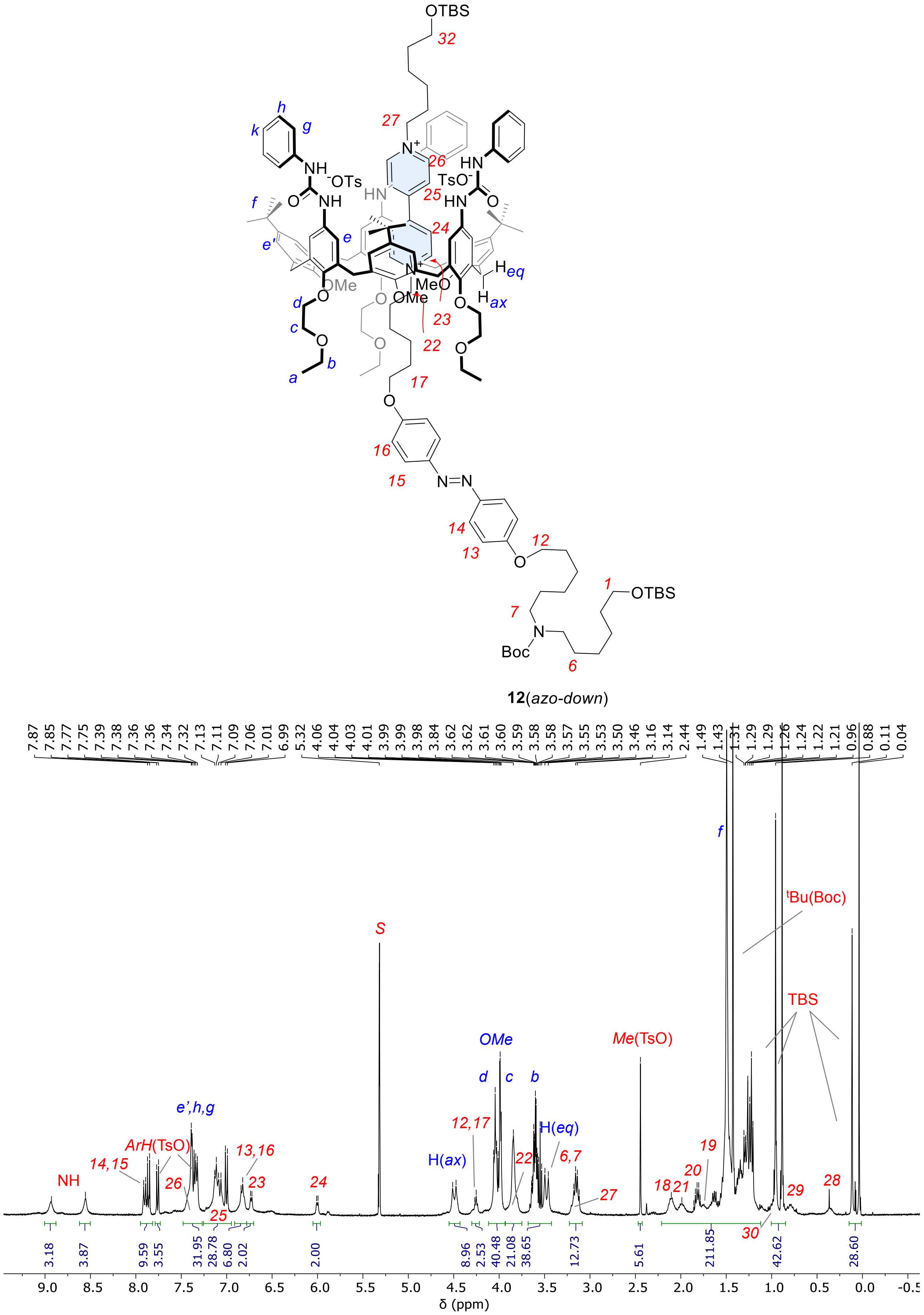

Figure S22. ^{1}H NMR spectrum (400 MHz, CD_2Cl_2, 298 K) of rotaxane **12**(*azo-down*).

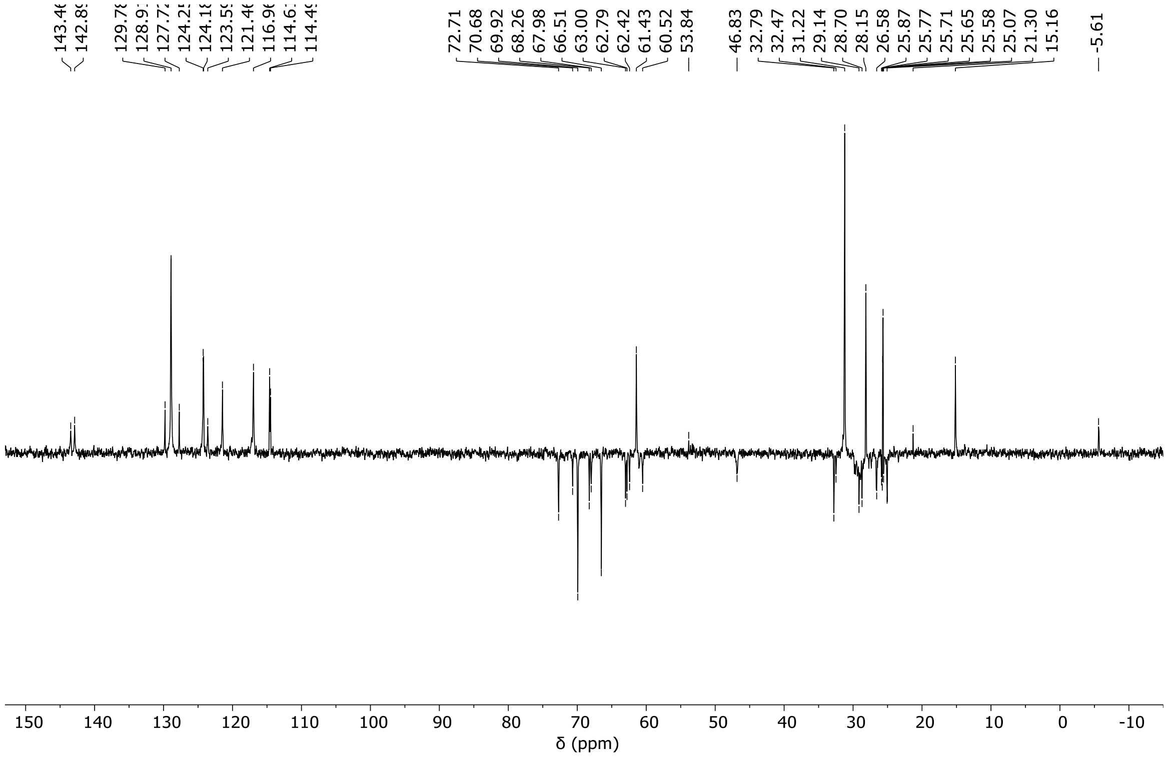

Figure S23. ^{13}C-APT NMR spectrum (100 MHz, CD_2Cl_2, 298 K) of rotaxane **12**(*azo-down*).

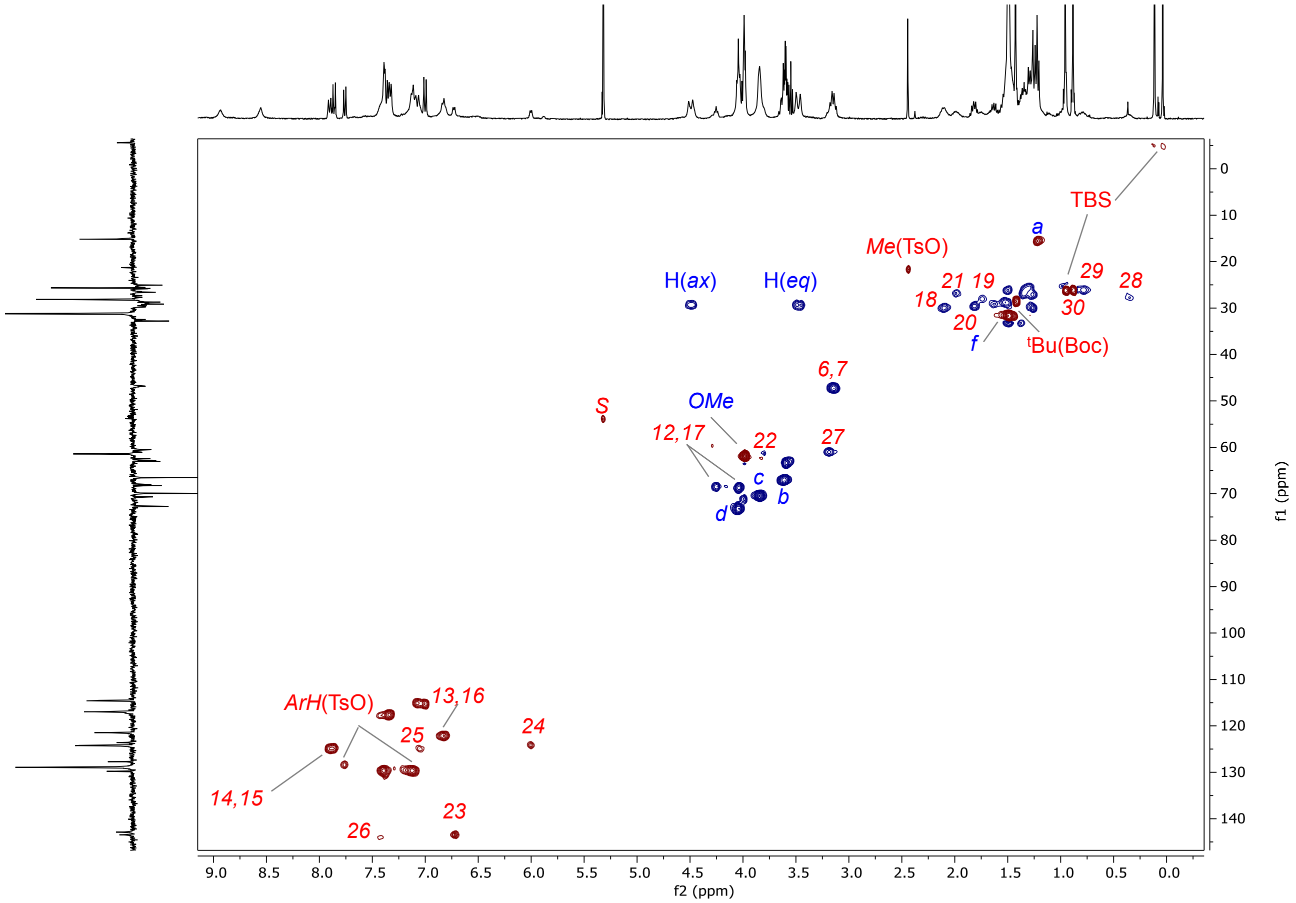

Figure S24. 2D Edited HSQC NMR spectrum (400 MHz, CD_2Cl_2, 298 K) of rotaxane **12**(*azo-down*). Positive peaks (CH_3 and CH) are shown in red, while negative ones (CH_2) are in blue.

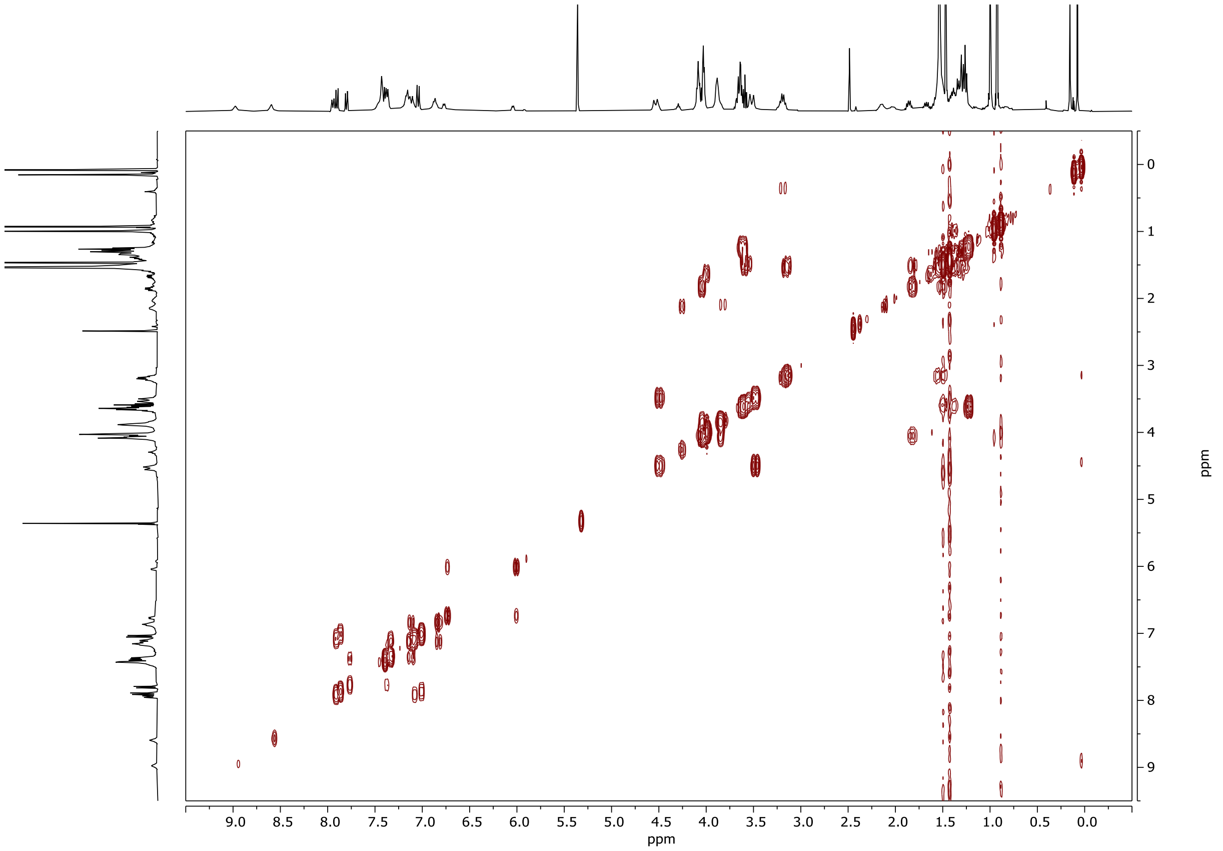

Figure S25. 2D COSY NMR spectrum (400 MHz, CD_2Cl_2, 298 K) of rotaxane **12**(*azo-down*).

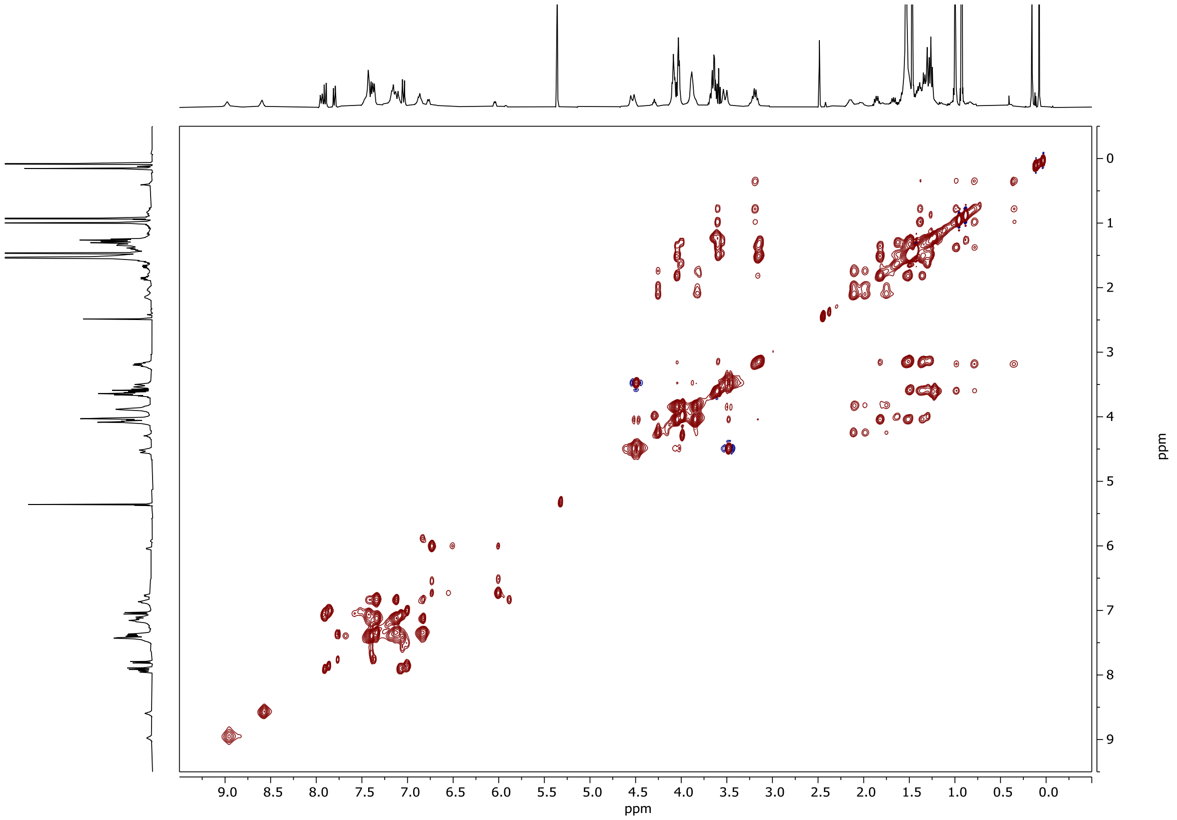

Figure S26. 2D TOCSY 2D NMR spectrum (400 MHz, CD_2Cl_2, 298 K, Mixing time = 0.04s)) of rotaxane **12**(*azo-down*).

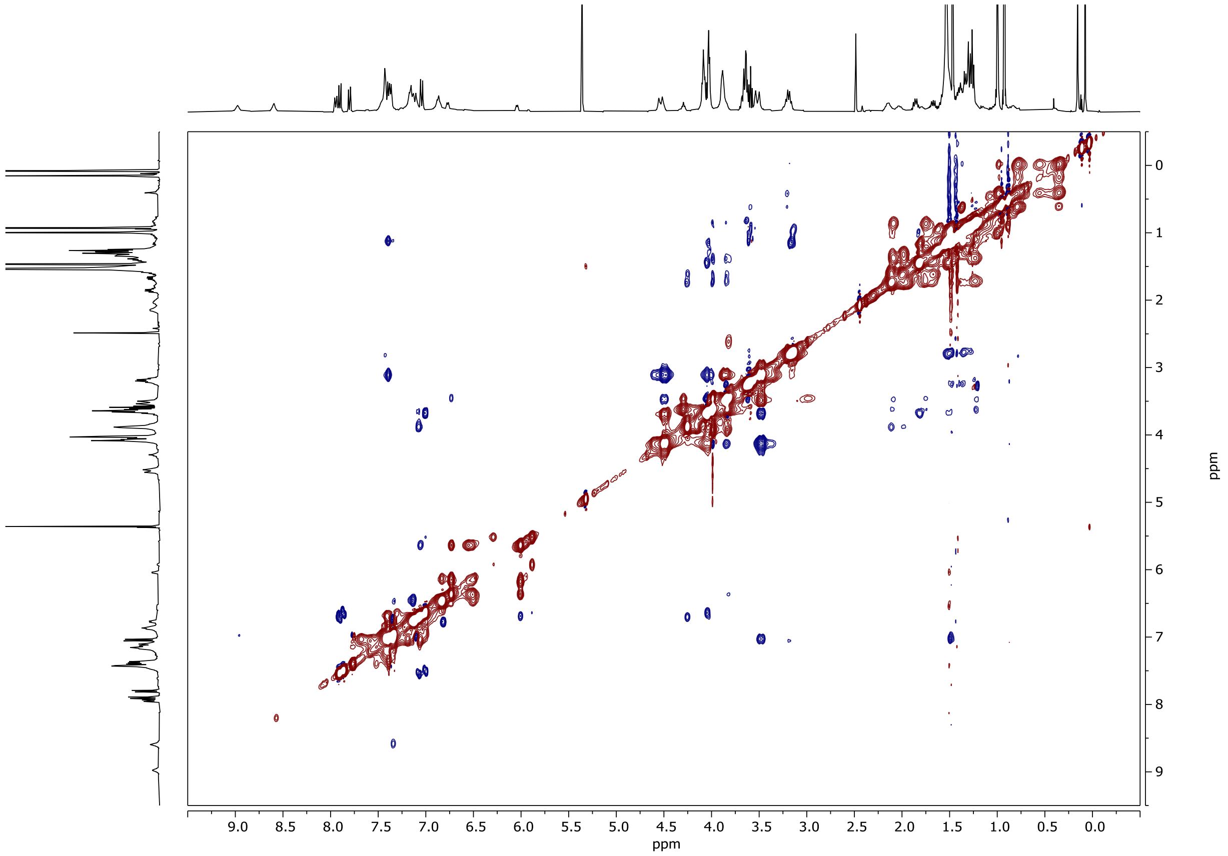

Figure S27. 2D NOESY NMR spectrum (400 MHz, CD_2Cl_2, 298 K, Spin-locking = 200 ms) of rotaxane **12**(*azo-down*).

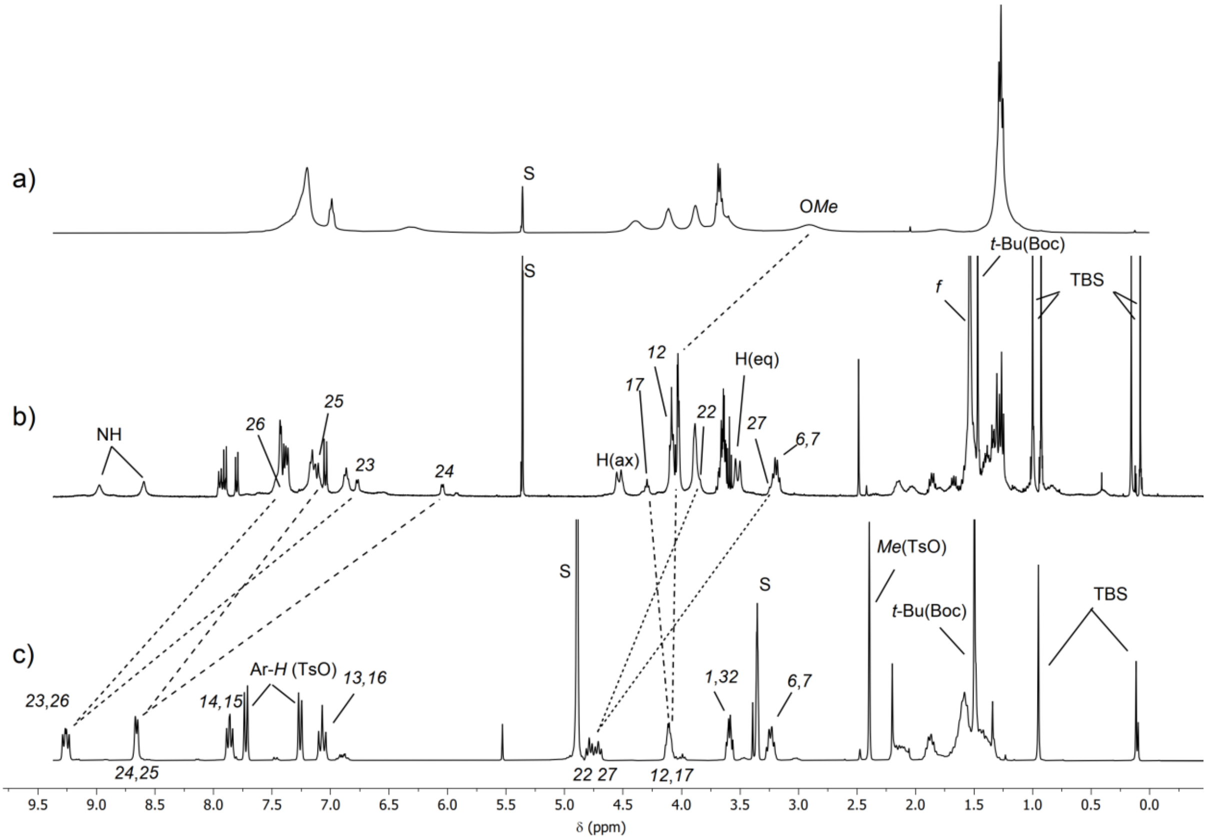

Figure S28. ^{1}H NMR stack plot (400 MHz) of a) **TPU**, b) rotaxane **12**(*azo-down*) in CD_2Cl_2, and c) axle **10** in CD_3OD. For the protons' labeling, see the sketch above.

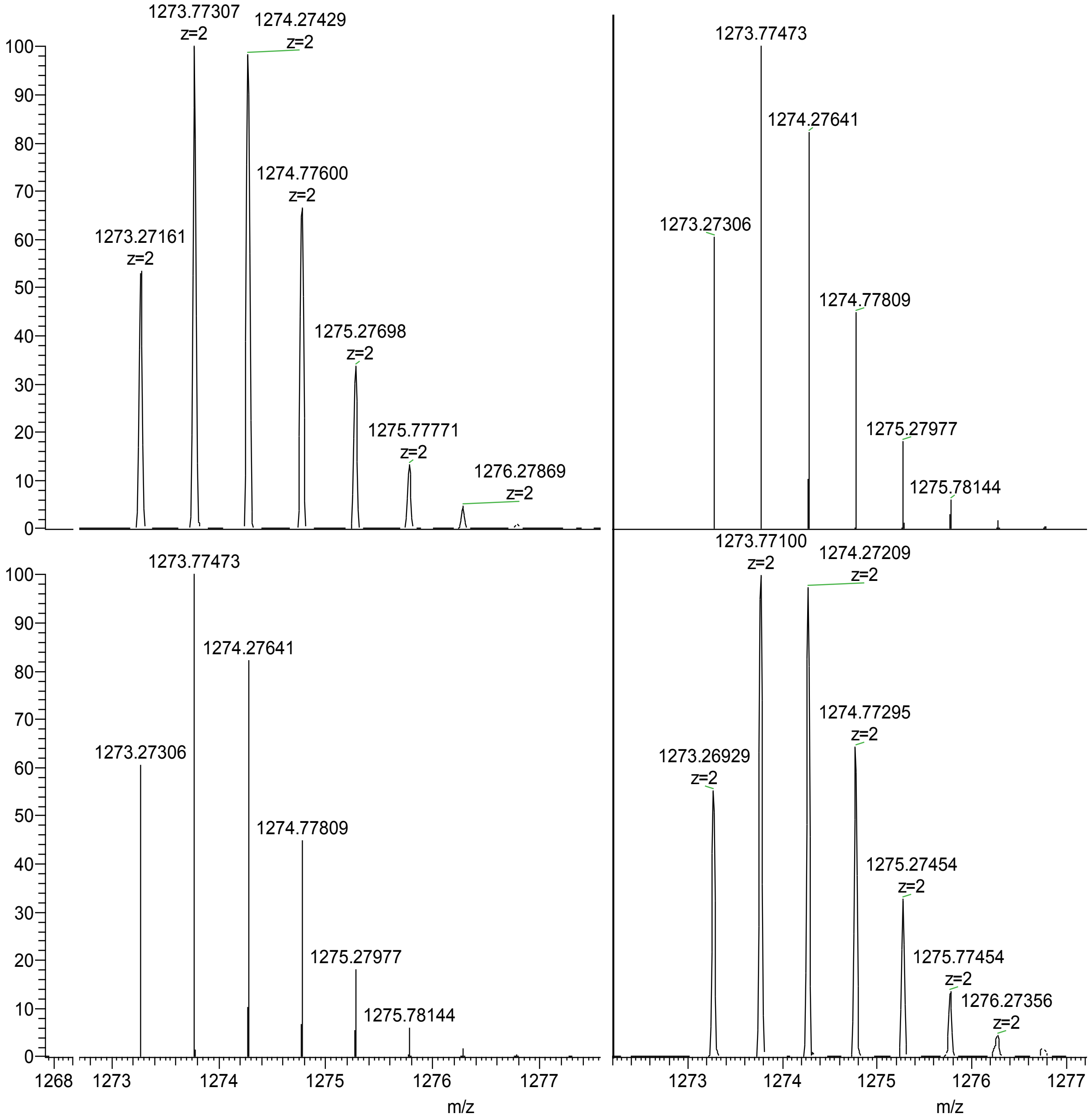

Figure S29. HR-MS spectra (ESI-ORBITRAP) of the rotaxane orientational isomers **12**(*azo-up*) (top left) and **12**(*azo-down*) (bottom right) with the corresponding theoretical isotopic distributions in the same column.

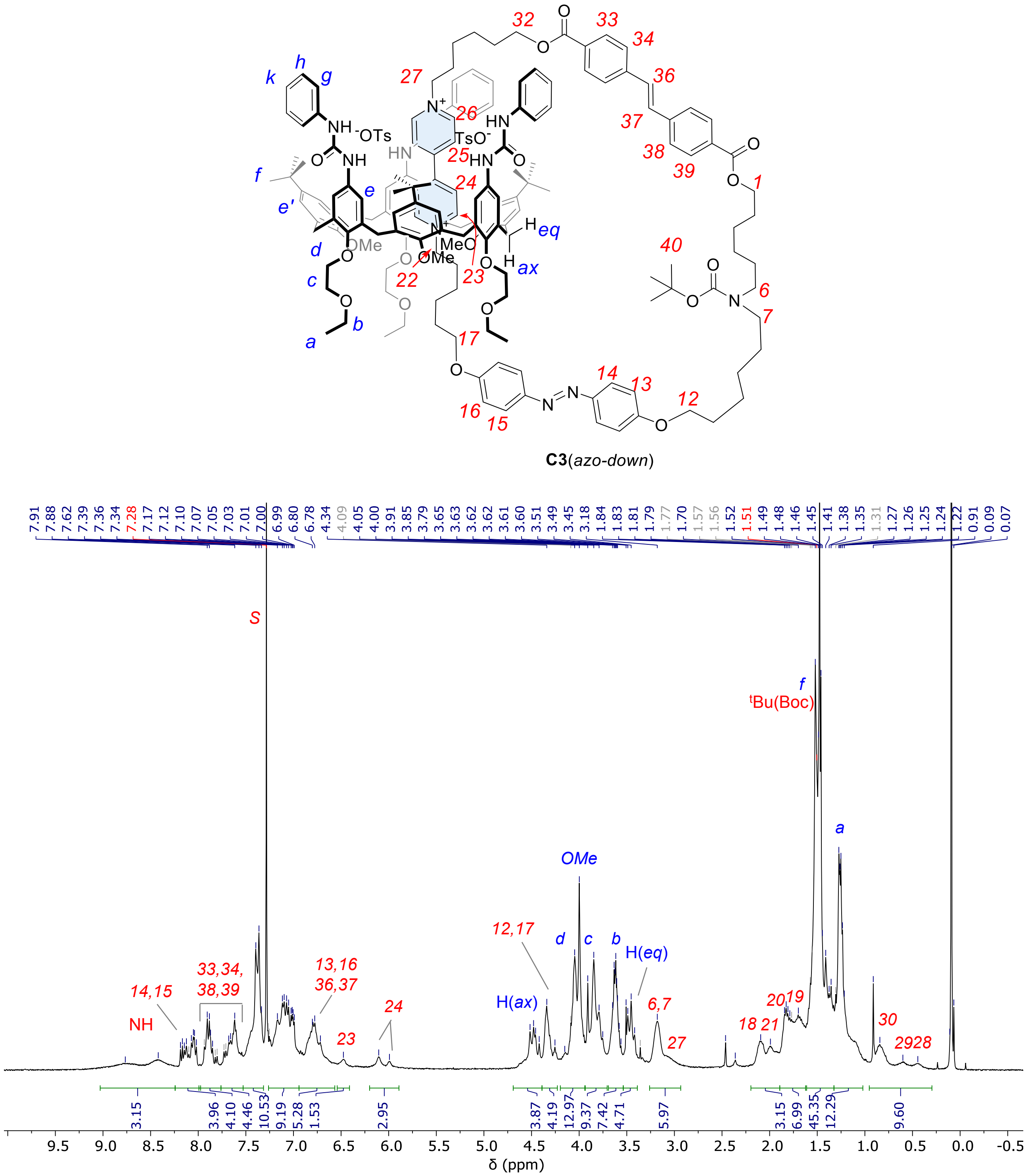

Figure S30. ^{1}H NMR spectrum (400 MHz, $CDCl_3$, 298 K) of catenane **C3**(*azo-down*).

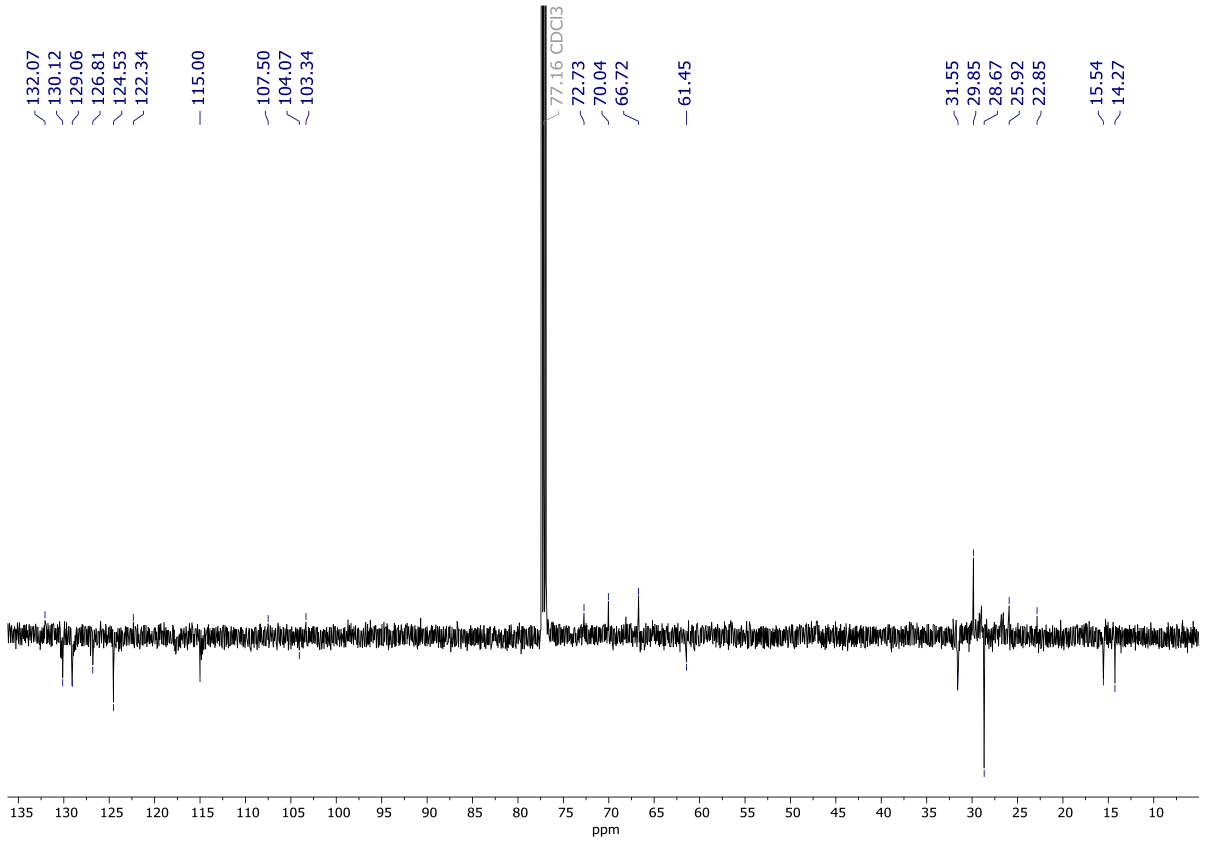

Figure S31. ^{13}C-DEPTQ NMR spectrum (150 MHz, $CDCl_3$, 298 K) of catenane **C3**(*azo-down*).

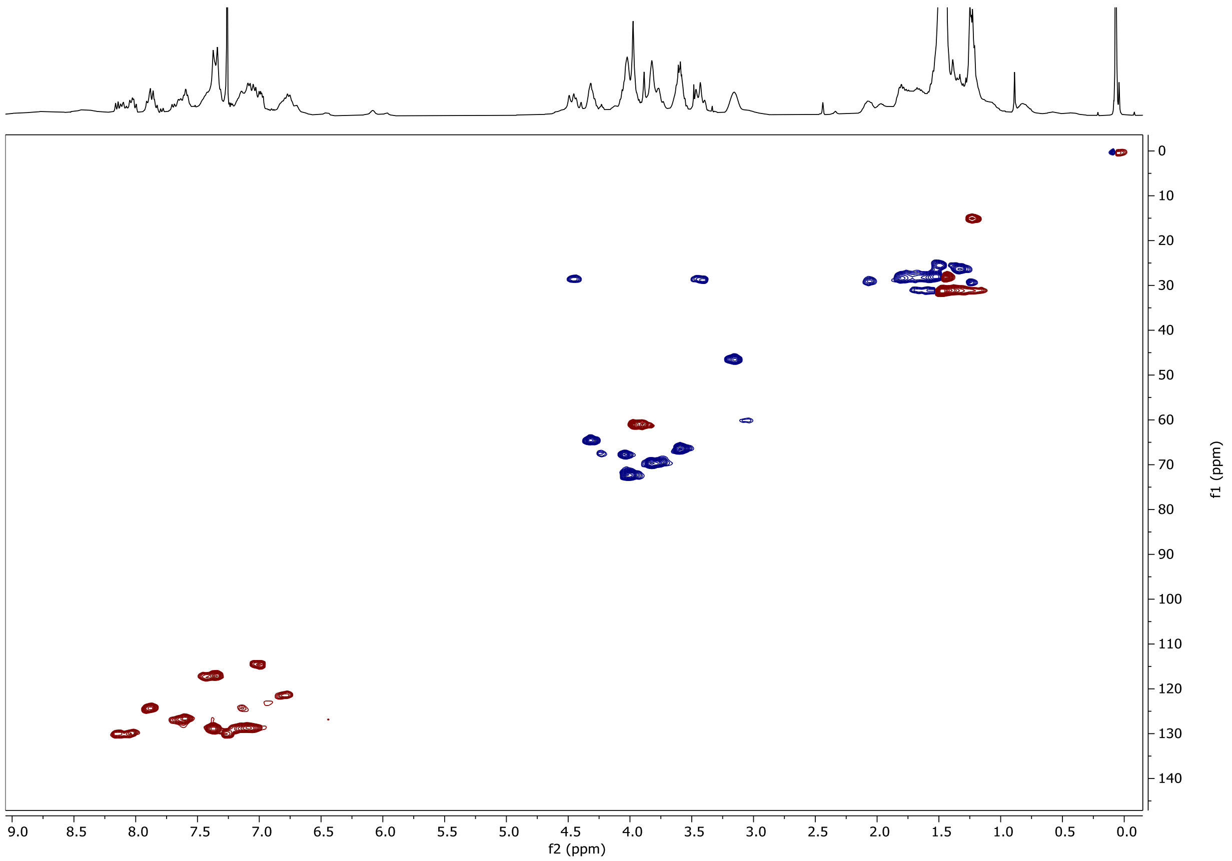

Figure S32. 2D Edited HSQC NMR spectrum (400 MHz, $CDCl_3$, 298 K) of catenane **C3**(*azo-down*). Positive peaks (CH_3 and CH) are shown in red, while negative ones (CH_2) are in blue.

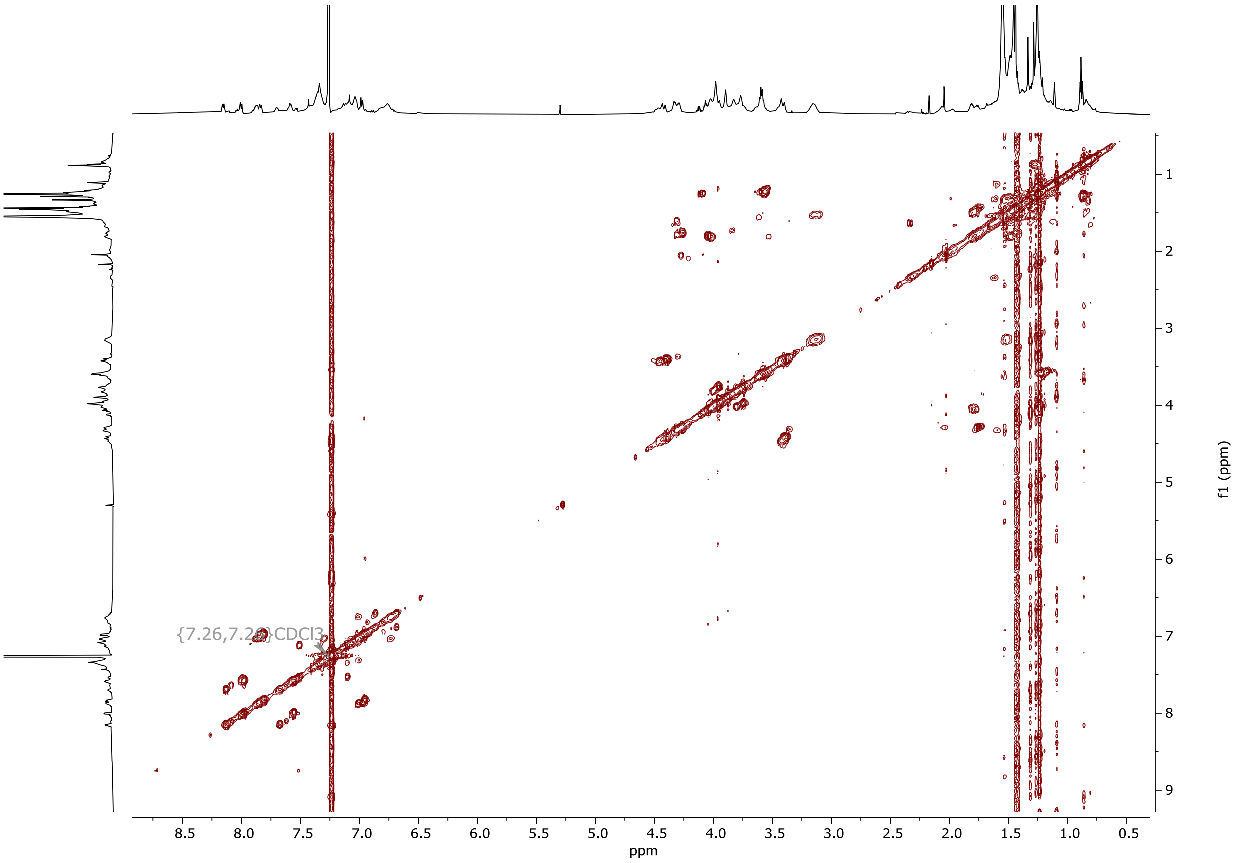

Figure S33. 2D COSY NMR spectrum (600 MHz, $CDCl_3$, 298 K) of catenane **C3**(*azo-down*).

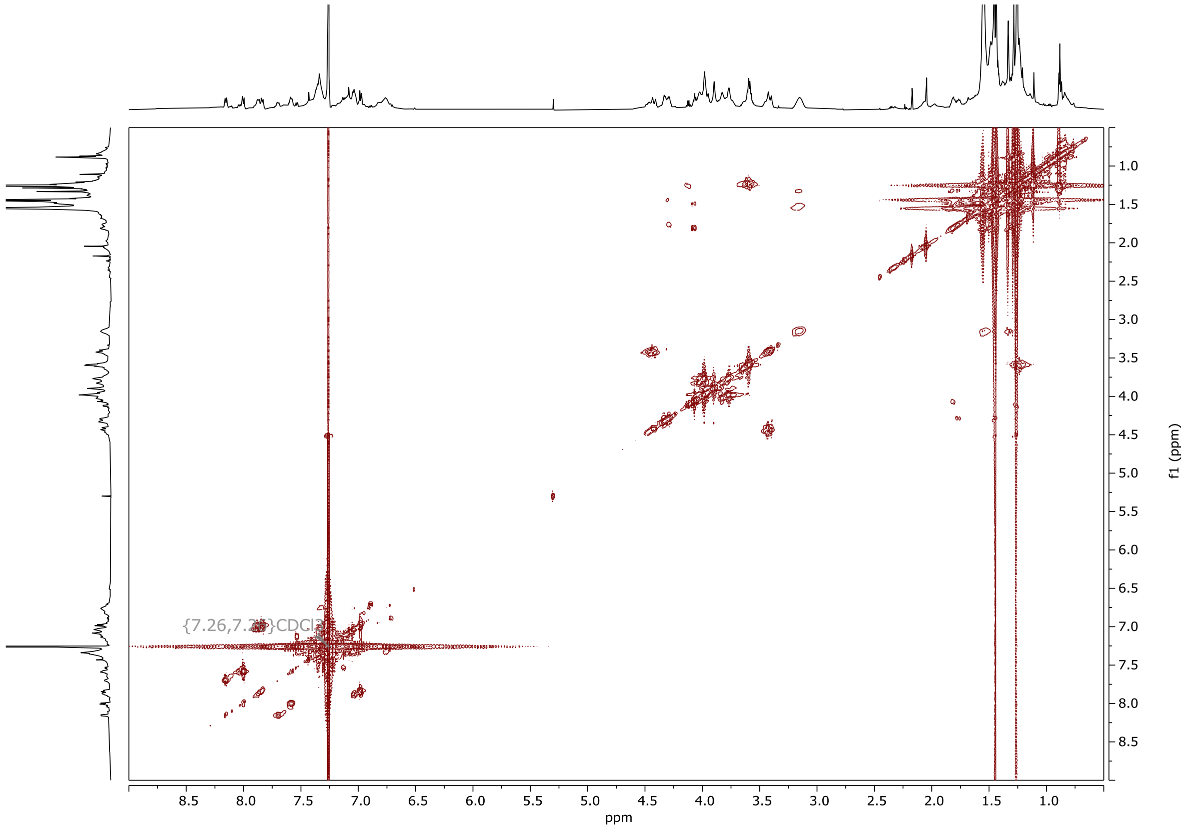

Figure S34. 2D TOCSY 2D NMR spectrum (600 MHz, $CDCl_3$, 298 K, Mixing time = 0.04s)) of catenane **C3**(*azo-down*).

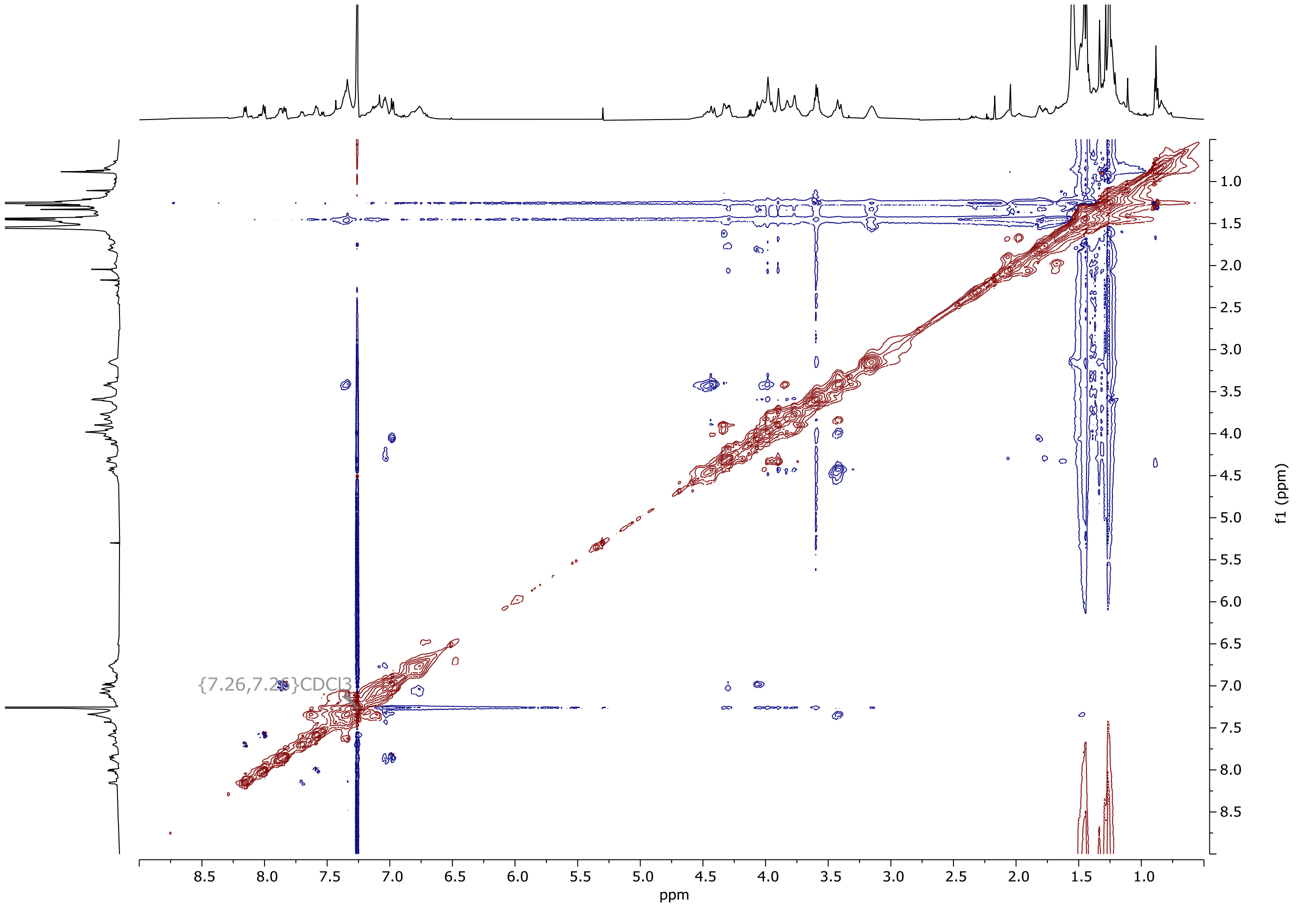

Figure S35. 2D ROESY NMR spectrum (600 MHz, $CDCl_3$, 298 K, Spin locking = 200 ms) of catenane **C3**(*azo-up*).
